# Dynamically linking influenza virus infection kinetics, lung injury, inflammation, and disease severity

**DOI:** 10.7554/eLife.68864

**Published:** 2021-07-20

**Authors:** Margaret A Myers, Amanda P Smith, Lindey C Lane, David J Moquin, Rosemary Aogo, Stacie Woolard, Paul Thomas, Peter Vogel, Amber M Smith

**Affiliations:** 1 Department of Pediatrics, University of Tennessee Health Science Center Memphis United States; 2 Department of Anesthesiology, Washington University School of Medicine St. Louis United States; 3 Flow Cytometry Core, St. Jude Children's Research Hospital Memphis United States; 4 Department of Immunology, St. Jude Children’s Research Hospital Memphis United States; 5 Department of Pathology, St. Jude Children's Research Hospital Memphis United States; Fred Hutchinson Cancer Research Center United States; University of Colorado Boulder United States

**Keywords:** influenza virus, mathematical model, lung injury, lung inflammation, disease severity, CD8 T cells, Virus

## Abstract

Influenza viruses cause a significant amount of morbidity and mortality. Understanding host immune control efficacy and how different factors influence lung injury and disease severity are critical. We established and validated dynamical connections between viral loads, infected cells, CD8^+^ T cells, lung injury, inflammation, and disease severity using an integrative mathematical model-experiment exchange. Our results showed that the dynamics of inflammation and virus-inflicted lung injury are distinct and nonlinearly related to disease severity, and that these two pathologic measurements can be independently predicted using the model-derived infected cell dynamics. Our findings further indicated that the relative CD8^+^ T cell dynamics paralleled the percent of the lung that had resolved with the rate of CD8^+^ T cell-mediated clearance rapidly accelerating by over 48,000 times in 2 days. This complimented our analyses showing a negative correlation between the efficacy of innate and adaptive immune-mediated infected cell clearance, and that infection duration was driven by CD8^+^ T cell magnitude rather than efficacy and could be significantly prolonged if the ratio of CD8^+^ T cells to infected cells was sufficiently low. These links between important pathogen kinetics and host pathology enhance our ability to forecast disease progression, potential complications, and therapeutic efficacy.

## Introduction

Over 15 million respiratory infections and 200,000 hospitalizations result from influenza A viruses (IAVs) each year (Thompson et al., 2004; Simonsen et al., 2000; Taubenberger and Morens, 2008; Medina and García-Sastre, 2011). The incidence and severity of IAV infections increases when new strains emerge and/or when there is a lack of prior immunity. A robust immune response is crucial for resolving viral infections, but immune-mediated pathology can exacerbate disease (Duan and Thomas, 2016; Moskophidis and Kioussis, 1998; Mauad et al., 2010; Rygiel et al., 2009; La Gruta et al., 2007). High viral loads can also play a role in disease progression (Boon et al., 2011; de Jong et al., 2006), but these do not always correlate with the strength of the host response or with disease severity (Granados et al., 2017; Marathe et al., 2016; Toapanta and Ross, 2009; Smith et al., 2019; Gao et al., 2013). An understanding of how viral loads, host immune responses, and disease progression are related is critical to identify disease-specific markers that may help predict hospitalization or other complications.

During IAV infection in both humans and animals, viral loads increase rapidly for the first 1–2 days of infection before reaching a peak (e.g., as in Smith et al., 2018; Baccam et al., 2006; Miao et al., 2010; Toapanta and Ross, 2009; Carrat et al., 2008; Srivastava et al., 2009; Gao et al., 2013). If the host is infected with a novel strain or has no prior immunity, viral loads in the lung then begin to decline, first slowly (sometimes <1 log_10_ TCID_50_/d ) then rapidly (> 4-5 log_10_ TCID_50_/d) (Smith et al., 2018; Gao et al., 2013). We previously quantified this biphasic viral decline with a mathematical model, which indicated that the rate of infected cell clearance increases as the density of infected cells decreases (Smith et al., 2018). The timing of the second, rapid viral decay phase coincides with the expansion of CD8^+^ T cells, which are the primary cell responsible for clearing infected cells and resolving the infection (Zhang and Bevan, 2011; Chen et al., 2018; McMichael et al., 1983; La Gruta and Turner, 2014; Kreijtz et al., 2011), and, to a lesser extent, neutralizing antibodies (Chen et al., 2018; Kreijtz et al., 2011; Fang and Sigal, 2005; Wang et al., 2015; McMichael et al., 1983) and cytotoxic CD4^+^ T cells (Wilkinson et al., 2012). For the CD8^+^ T cell response, in particular, it remains unclear whether the efficacy of these cells is dictated by their own density (Graw and Regoes, 2009; Wiedemann et al., 2006), infected cell density (Merrill, 1982; Caramalho et al., 2009; Halle et al., 2016), or both (Gadhamsetty et al., 2014). While quantifying dynamically changing CD8^+^ T cell efficacy is difficult *in vitro* and *in vivo*, the question is ripe for *in silico* investigation. Several modeling studies have described CD8^+^ T cell-mediated infected cell clearance for various viral infections in humans and animals, including IAV, HIV, and LCMV (e.g., as in Ganusov et al., 2011; De Boer and Perelson, 1998; Perelson and Bell, 1982; Miao et al., 2010; Cao et al., 2016; Lee et al., 2009; Price et al., 2015; Graw and Regoes, 2009; Merrill, 1982; Gadhamsetty et al., 2014; Baral et al., 2019; Bonhoeffer et al., 2000; Conway and Perelson, 2015). Some of these studies have also attempted to link this response and other immune responses to inflammation or disease severity (Price et al., 2015; Baral et al., 2019; Manchanda et al., 2014), but have not yet found the appropriate mathematical relation with the available data. In addition, for IAV infections in particular, varied efficiency of the CD8^+^ T cell response throughout the course of infection, their early antigen-specific and lung-resident responses (Yoon et al., 2007; McGill and Legge, 2009; Keating et al., 2018), and the balanced consequences on viral loads and host pathology have not yet been investigated in detail.

A better understanding of infected cell clearance may also yield insight into the damage induced to the lung and the ensuing immunopathology during IAV infection. In general, widespread alveolar disease is observed in patients who succumb to the infection (Bautista et al., 2010; Shieh et al., 2010; Gao et al., 2013), and CT scans show bilateral and multi-focal areas of lung damage (e.g. as in Kohr et al., 2010; Li et al., 2011; Ajlan et al., 2009; Soto-Abraham et al., 2009; Gill et al., 2010; Li et al., 2012). Further, hospitalized patients that died as a result from infection with the 2009 H1N1 influenza virus had large numbers of CD8^+^ T cells present in their lung tissue (Mauad et al., 2010). Large pulmonary populations of CD8^+^ T cells contribute to lung injury by targeting IAV-infected cells (reviewed in Duan and Thomas, 2016) and inducing ‘bystander damage’ to uninfected epithelial cells (van de Sandt et al., 2017). However, their population levels do not necessarily indicate active infected cell removal or immunopathology as their extended presence in the lung also contributes to surveillance and repair (Matheu et al., 2013; Sun et al., 2009). Macrophages and neutrophils also persist in the lung following viral clearance, and their role in inflammation and tissue damage is well documented (reviewed in Watanabe et al., 2019; La Gruta et al., 2007). A favorable outcome requires a balance between immune-mediated protection and pathology, and the viral-immunological landscape that drives disease and the markers that distinguish mild from severe influenza are complex. This is particularly true in humans (Tang et al., 2019) where obtaining high quality data from the lung is challenging, and viral loads in the upper respiratory tract do not always reflect the lower respiratory tract environment (Feikin et al., 2017; Ai et al., 2020; Wong et al., 2020; Li et al., 2012; Ruuskanen et al., 2011).

The accumulation of damage to the epithelium during IAV infection, either from virally-induced cell lysis or immune-mediated effects, is relatively understudied. We and others have modeled the lung damage and inflammation inflicted during pulmonary infections (e.g., as in Smith et al., 2011b; Reynolds et al., 2006; Dunster et al., 2014; Ramirez-Zuniga et al., 2019; Baral et al., 2019) but did so without sufficient data to constrain the model formulations. The difficulty in measuring the dynamics of infected cells and in establishing how damage correlates to specific host responses has been the primary impediment. However, recent technological advances, including the use of reporter viruses (Manicassamy et al., 2010; Tran et al., 2013; Karlsson et al., 2015; Luker and Luker, 2010) and lung histomorphometry (Marathe et al., 2016; Marathe et al., 2017; Sartorius et al., 2007; Zachariadis et al., 2006; Boyd et al., 2020) within animal models, have provided an opportunity to acquire these types of measurements. Whole lung histomorphometry, which is broadly defined as a quantitative microscopic measurement of tissue shape, has recently increased in use due to the ability to directly stain, visualize, and quantify areas of infected cells. Even with these techniques, quantitative data over the course of infection is not currently available. Having data such as these should help reconcile potential nonlinearities in infected cell clearance and provide insight into the accumulated lung inflammation and damage, which are generally thought to be markers of disease severity. In addition, it should bolster the development of robust, predictive computational models, which have historically lacked constraint to these types of data.

In general, disease severity may not be directly correlated to viral loads or specific immunological components. In humans infected with IAV, viral loads typically increase prior to the onset of systemic symptoms, which peak around 2–3 d post-infection (pi) (Carrat et al., 2008; Lee and Lee, 2010; Han et al., 2018). Some symptoms (e.g., fever) are cytokine mediated (Monto et al., 2000), but respiratory symptoms often last longer and can remain following viral clearance (Lee and Lee, 2010; Eccles, 2005). This is particularly true when there is scarring of the lung tissue (Li et al., 2012). In the murine model, weight loss is used as an indicator of disease progression and severity, where greater weight loss corresponds to more severe disease (Trammell and Toth, 2011; Bouvier and Lowen, 2010; Parzych et al., 2013). Animal weights generally drop slowly in the first several days during an IAV infection and more rapidly as the infection begins to resolve (Srivastava et al., 2009; Huang et al., 2017). This is unlike viral load dynamics in these animals, which increase rapidly during the first 0–3 d pi then remain relatively constant prior to resolution (Smith et al., 2018). Because weight loss can occur following resolution of the infection, immune-mediated pathology is thought to play a role (Lauder et al., 2011; Xu et al., 2004; Ostler et al., 2002; Parzych et al., 2013; Duan and Thomas, 2016). Host and pathogen factors, such as age, viral proteins, and inoculum size, have also been shown to influence disease progression (Toapanta and Ross, 2009; Lu et al., 2018; Smith et al., 2019; McAuley et al., 2007). While the precise contribution of different factors to IAV-associated disease and mortality remain elusive, having tools that can link these with disease severity and decipher correlation from causation would improve our ability to effectively predict, understand, and treat the disease.

To gain deeper insight into the dynamics of viral resolution during primary pulmonary influenza infection and investigate the connection between viral loads, damage, inflammation, and disease severity, we developed and validated a mathematical model that describes viral kinetics and CD8^+^ T cell-mediated infected cell clearance. The model accurately predicted the dynamics of effector and memory CD8^+^ phenotypes, agreed with our previous results that infected cells are cleared in a density-dependent manner with CD8 efficiency rapidly accelerating by over 48,000 times in 2 d, and illuminated tradeoffs between the innate and adaptive immune responses. Our model predicted that infection duration is dependent on the magnitude of CD8^+^ T cells rather than their efficacy, which we verified by depleting CD8^+^ T cells. Quantitative whole lung histomorphometry showed that infected areas increase during the first 6 d of infection before clearing rapidly, that the relative number of CD8^+^ T cells corresponded to the cleared area, and corroborated the model-predicted infected cell dynamics. These data also showed that the infection-induced lung injury is distinct from inflammation, and that each correlates to different immune responses and could be predicted using independent mathematical equations. In addition, the data revealed nonlinear connections between these two pathological measurements and disease severity. Together, our data, model, and analyses provide a robust quantification of the density-dependent nature of CD8^+^ T cell-mediated clearance, and the critical connections between these cells and the dynamics of viral loads, infected cells, lung injury, inflammation, and disease severity.

## Results

### Virus and CD8^+^ T cell kinetics

**Figure 1.**
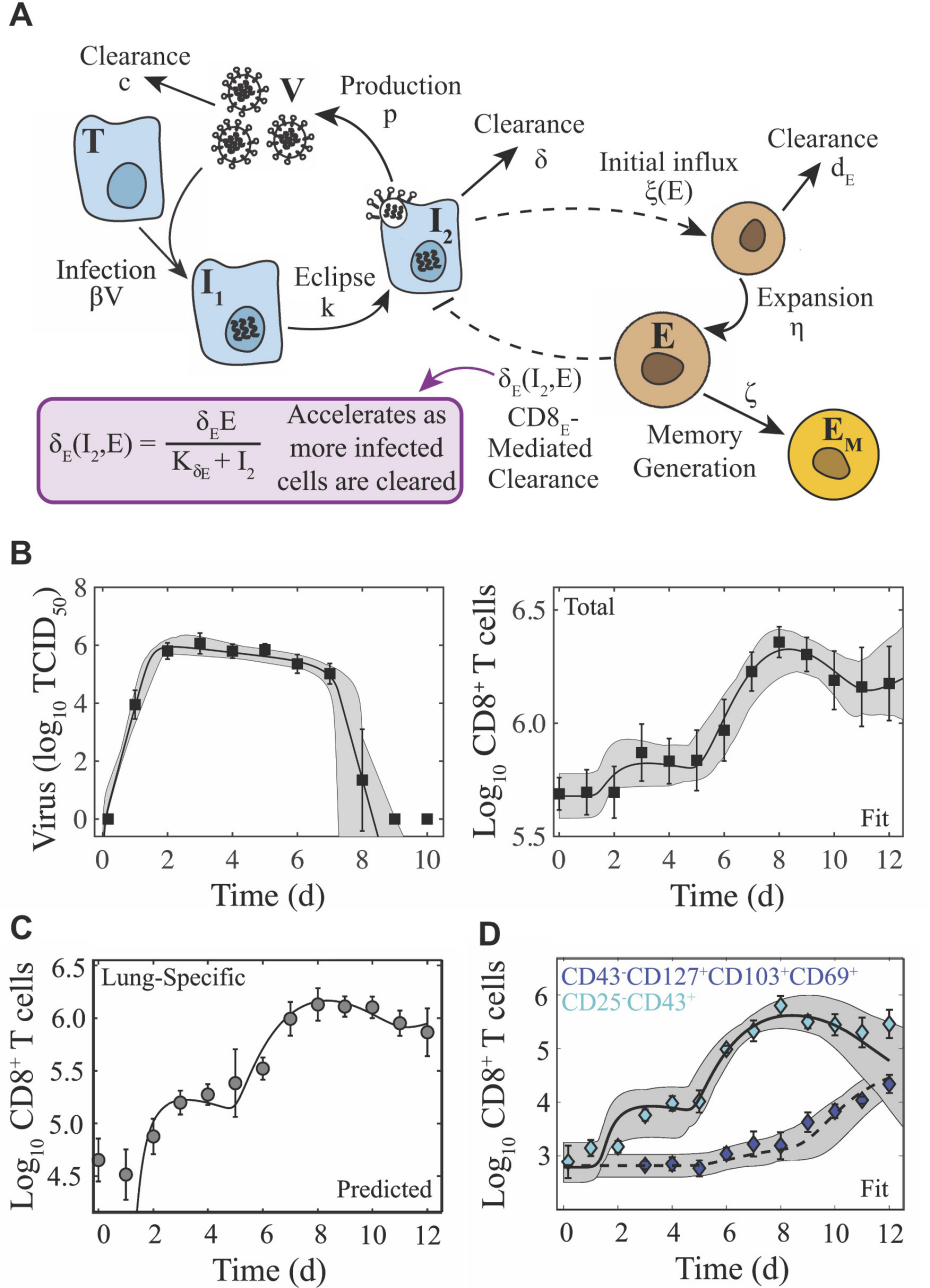
Schematic and fit of the CD8^+^ T cell viral kinetic model. (**A**) Schematic of the CD8^+^ T cell model in Equation (1)-(6). In the model, target cells (T) are infected at rate β⁢V. Infected cells ($I_{1}$) undergo an eclipse phase and transition to become productively-infected cells ($I_{2}$) at rate k. Virus (V) is produced by infected cells at rate p and is cleared at rate c. Infected cells are cleared at rate δ by non-specific mechanisms and at rate $\delta_{E}\,\left(I_{2},E\right)$ by effector CD8^+^ T cells (E; denoted CD8_E_). The dashed lines represent interactions between infected cells and CD8_E_. Initial CD8_E_ influx ($\xi \,\left(E\right)=\xi /\left(K_{E}+E\right)$) is proportional to infected cells and is limited by CD8_E_ with half-saturation constant $K_{E}$. CD8_E_ expansion (η) occurs proportional to infected cells $\tau_{E}$ days ago. Memory CD8^+^ T cell ($E_{M}$; denoted CD8_M_) generation occurs at rate ζ and proportional to CD8_E_ $\tau_{M}$ days ago. (**B**) Fit of the CD8^+^ T cell model (Equation (1)-(6)) to virus and total CD8^+^ T cells from the lungs of mice infected with 75 TCID_50_ PR8 (10 mice per time point). The total number of CD8^+^ T cells is $\hat{E}=E+E_{M}+{\hat{E}}_{0}$. (**C**) Total CD8^+^ T cells in the lung parenchyma (gray circles) and overlay of the model predicted values ($E+E_{M}$). (**D**) Fit of the model to virus, CD25^–^CD43^+^ CD8^+^ T cells (cyan diamonds; E), and CD43^–^CD127^+^CD103^+^CD69^+^ CD8^+^ T cells (blue diamonds; $E_{M}$) (five mice per time point). The solid and dashed black lines are the optimal solutions and the gray shading is are the model solutions using parameter sets within the 95% CIs. Parameters are given in Table 1. Data are shown as mean ± standard deviation.

In animals infected with 75 TCID_50_ PR8, virus rapidly infects cells or is cleared within 4 hr pi and is undetectable in all animals at this time (Figure 1). Virus then increases exponentially and peaks after ∼2 d pi. Following the peak, virus enters a biphasic decline. In the first phase (2–6 d pi), virus decays slowly and at a relatively constant rate (0.2 log_10_ TCID_50_/d) (Smith et al., 2018). In the second phase (7–8 d pi), virus is cleared rapidly with a loss of 4–5 log_10_ TCID_50_ in 1–2 d (average of –3.8 log_10_ TCID_50_/d) (Smith et al., 2018). CD8^+^ T cells remain at their baseline level from 0–2 d pi, where ∼15% are located in the circulating blood, ∼75% in the lung vasculature, and ∼10% in the lung parenchyma (Figure 1—figure supplement 1) with the majority in the lung as CD43^–^CD127^+^. IAV-specific CD8^+^ T cells that are recently primed (CD25^+^CD43^+^) or effector cells (CD25^–^CD43^+^) (Keating et al., 2018) begin appearing in the lung and increase slightly from 2–﻿3 d pi but remain at low levels. These cells are thought to proliferate within the lung at least once by 4 d pi (McGill and Legge, 2009), and their populations briefly contract (3–5 d pi) before expanding rapidly (5–8 d pi). During the expansion phase, >95% of recovered cells are in the lung (∼70% in the parenchyma and ∼30% in the vasculature) with CD25^+^CD43^+^ and CD25^–^CD43^+^ as the predominant phenotypes. This is in accordance with prior studies that showed these phenotypes are IAV-specific and that their expansion dynamics in the lung were not altered by removing blood-borne CD8^+^ T cells from the analysis (Anderson et al., 2012; Keating et al., 2018).

**Figure 1—figure supplement 1.**
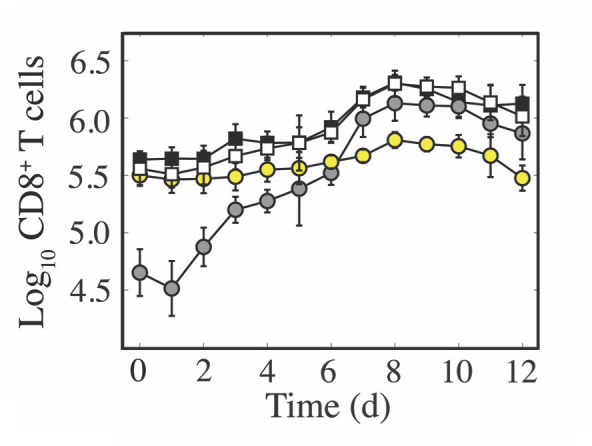
Dynamics of CD8^+^ T cells in the lung parenchyma and vasculature. Comparison of total CD8^+^ T cells in a non-perfused lung (black) and in a perfused lung (white). Of those from a perfused lung, comparison of CD8^+^ T cells located in the lung parenchyma (CD45^–^; gray) and vasculature (CD45^+^; yellow). These data show that perfusion does not alter the number of cells recovered, and that the total number of cells primarily reflects changes in the lung parenchyma with little fluctuation in the lung vasculature. The cells in the lung vasculature may be slightly overestimated late in the infection due to incomplete perfusions during the height CD8-mediated clearance (7–9 d pi) when the lung integrity is low.

**Figure 1—figure supplement 2.**
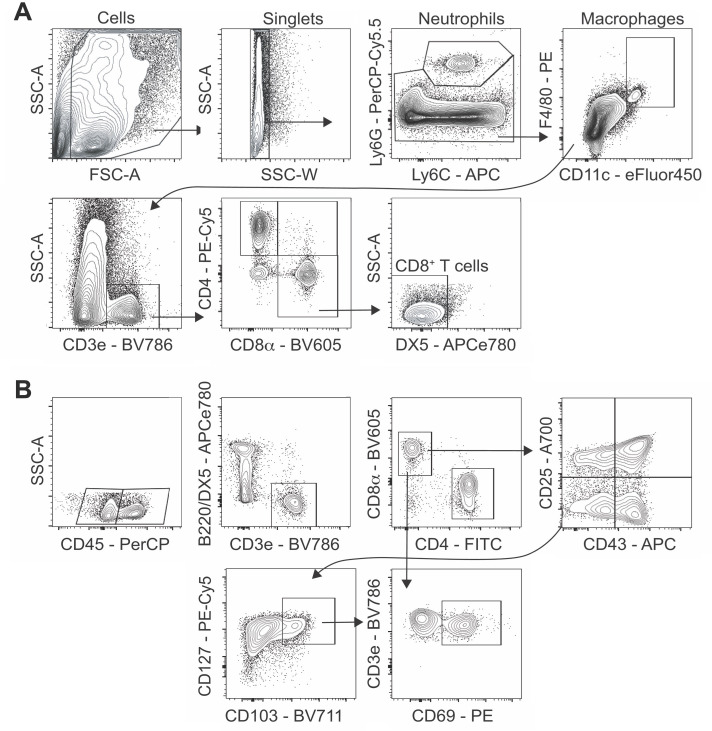
Flow cytometry gating strategy for CD8^+^ T cell analysis. Live cells were first gated on forward scatter (FSC-A) and side scatter (SSC-A) then as singlets. (**A**) Following neutrophil (Ly6G^hi^) and macrophage (F4/80^hi^ CD11c^hi^) exclusion, T cells were gated as CD3e^+^ with total CD8^+^ T cells sub-gated as DX5^–^CD4^–^CD8^+^. (**B**) Blood borne CD8^+^ T cells were gated as CD45^+^ cells and those in the lung parenchyma as CD45^–^ with the total in each sub-population gated as CD3e^+^B220^–^DX5^–^CD4^–^CD8^+^. IAV-specific CD8^+^ T cells were sub-gated as CD25^+^CD43^+^ (recently activated), CD25^–^CD43^+^ (effector), and CD25^–^CD43^–^CD127^+^CD103^+^CD69^+^ (long-lived memory). Expression of CD44, CD69, CD62L, and NKG2D were also assessed to ensure appropriate classification.

CD8^+^ T cell expansion corresponds to the second viral decay phase with sixty percent of mice clearing the infection by 8 d pi and the other forty percent by 9 d pi (Figure 1). Most CD43^+^CD8^+^ T cell phenotypes decline following viral clearance (8–10 d pi) but do not return to their baseline level by 12 d pi. Long-lived antigen-specific memory phenotypes down regulate CD43 (Jones et al., 1994; Harrington et al., 2000; Onami et al., 2002) and gradually increase substantially beginning at 9 d pi with most as CD25^–^, which is qualitatively similar to other studies (Harrington et al., 2000). At 12 d pi, ∼55% of CD8^+^ T cells remain in the lung parenchyma and ∼20% in the circulating blood indicating exit from the lung (Figure 1—figure supplement 1).

### Viral kinetic model with density-dependent CD8^+^ T cell-mediated clearance

**Figure 2.**
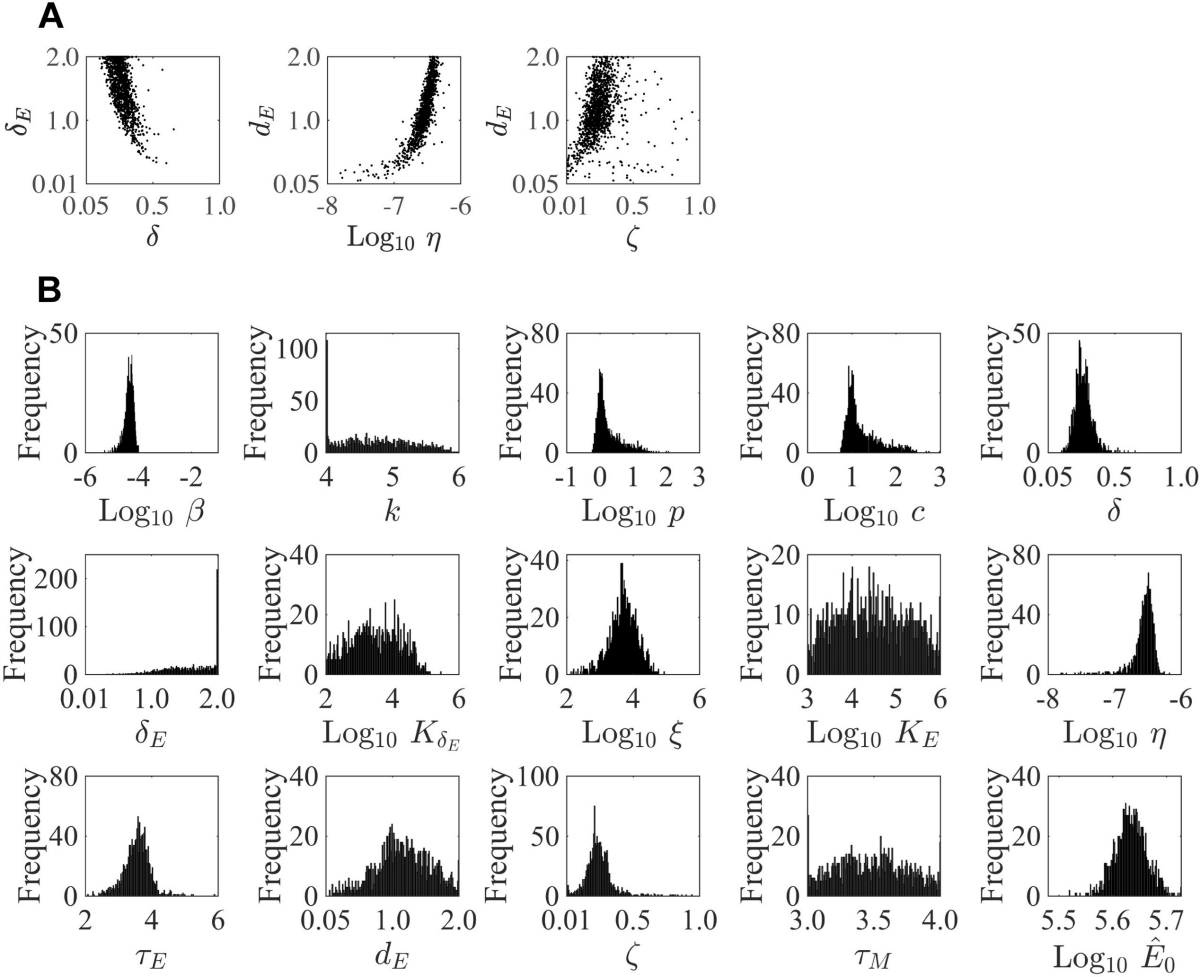
Parameter ensembles and histograms. Parameter ensembles (**A**) and histograms (**B**) resulting from fitting the CD8^+^ T cell viral kinetic model (Equation (1)-(6)) to viral titers and total CD8^+^ T cells from mice infected with 75 TCID_50_ PR8. (**A**) The rates of infected cell clearance by non-specific mechanisms (δ) and by CD8_E_ ($\delta_{E}$) are slightly negatively correlated. Correlations were also present between the rates of CD8_E_ clearance ($d_{E}$), CD8_E_ expansion (η), and CD8_M_ generation (ζ). The axes limits reflect imposed bounds. Additional ensemble plots are in Figure 2—figure supplements 1–2. (**B**) The histograms show that the majority of parameters are well-defined with the exception of the eclipse phase transition rate (k), one of the half-saturation constants ($K_{E}$), and the CD8_M_ generation delay ($\tau_{M}$).

We previously described the influenza viral load kinetics and biphasic decline during primary pulmonary infection using a density-dependent (DD) model (Smith et al., 2018), which assumes that the rate of infected cell clearance increases as the density of infected cells decreases (i.e., $\delta_{d}\,\left(I_{2}\right)=\delta_{d}/\left(K_{\delta }+I_{2}\right)$). Because the rapid decay of virus is thought to be due to the clearance of infected cells by CD8^+^ T cells, it is unknown if early CD8^+^ T cell presence contributes to infected cell clearance, and no model has captured the entire CD8^+^ T cell time course, we developed a model that describes the dynamics of these cells and their efficiency in resolving the infection (Equation (1)-(6); Figure 1A). The model includes equations for effector (E, denoted CD8_E_) and memory ($E_{M}$, denoted CD8_M_) CD8^+^ T cells, and two mechanisms of infected cell clearance. The first mechanism is from unspecified, innate mechanisms, which is relatively constant (δ) and primarily acts during the first viral decay phase (2–6 d pi). The second is the CD8_E_-mediated infected cell clearance, which occurs at a rate that increases as the density of infected cells decreases ($\delta_{E}\,\left(I_{2},E\right)=\delta_{E}\,E/\left(K_{\delta_{E}}+I_{2}\right)$) and primarily acts during the rapid, second viral decay phase (7–8 d pi). Excluding this density dependence entirely resulted in a significant and premature decline in viral loads, which disagreed with the experimental data. We also tested whether the density dependence could be included in the CD8^+^ T cell expansion rate rather than in the infected cell clearance rate (see Appendix 1) as other models have done (e.g., as in Baral et al., 2019; Bonhoeffer et al., 2000; Conway and Perelson, 2015). This modification yielded a close fit to the CD8^+^ T cell data at 6–10 d pi but not at the early time points (Appendix 1—figure 1). In addition, the viral load data was underestimated at 7 d pi causing the solution to miss the rapid decline between 7–8 d pi and result in a statistically poorer fit. Thus, retaining the density-dependence in the rate of infected cell clearance most accurately captured the entire dataset. The model includes terms for the initial CD8_E_ influx at 2–3 d pi ($\xi \,I_{2}/\left(K_{E}+E\right)$) and for CD8_E_ expansion ($\eta \,E\,I_{2}\,\left(t-\tau_{E}\right)$), which accounts for the larger increase between 5–8 d pi. To capture the contraction of CD8^+^ T cells between these times (3–5 d pi), it was necessary to assume that the initial CD8_E_ influx is regulated by their own population (i.e., $\xi \,\left(E\right)=\xi /\left(K_{E}+E\right)$). In both terms, the increase is proportional to the number of infected cells. Although memory CD8^+^ T cells were not the primary focus here, it was necessary to include the CD8_M_ population because CD8^+^ T cells are at a significantly higher level at 10–12 d pi than at 0 d pi (Figure 1B). Fitting the model simultaneously to viral loads and total CD8^+^ T cells from the (non-perfused) lungs of infected animals illustrated the accuracy of the model (Figure 1B). The resulting parameter values, 95% confidence intervals (CIs), ensembles, and histograms are given in Table 1, Figure 2, and Figure 2—figure supplements 1–2.

**Figure 2—figure supplement 1.**
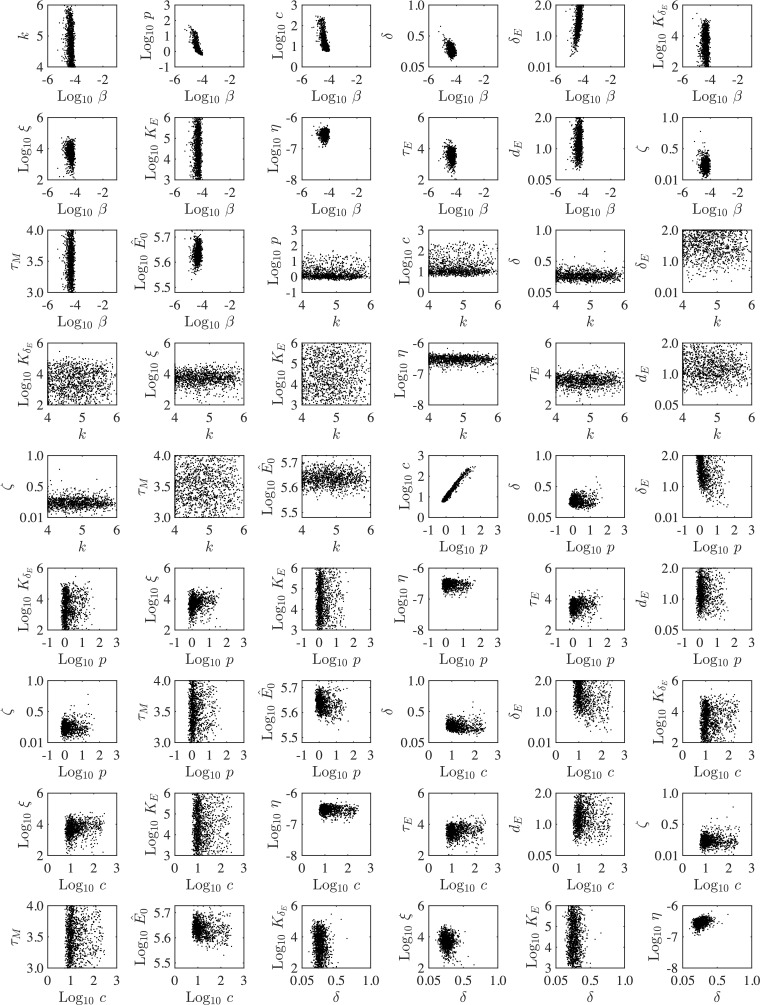
Parameter ensembles. Parameter ensembles resulting from fitting the CD8^+^ T cell model (Equation (1)-(6), Main Text) to viral loads and CD8^+^ T cells from mice infected with 75 TCID_50_ PR8. The axes limits reflect the imposed bounds. Additional ensemble plots are in Figure 2 (Main Text) and Figure 2—figure supplement 2.

**Figure 2—figure supplement 2.**
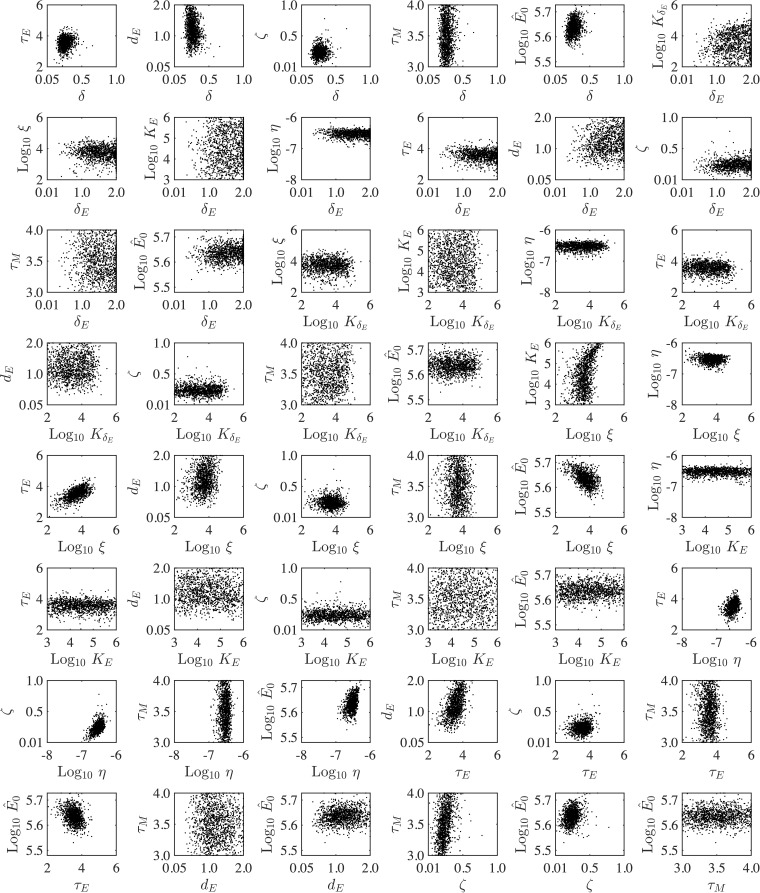
Parameter ensembles. Parameter ensembles resulting from fitting the CD8^+^ T cell model (Equation (1)-(6), Main Text) to viral loads and CD8^+^ T cells from mice infected with 75 TCID_50_ PR8. The axes limits reflect the imposed bounds. Additional ensemble plots are in Figure 2 (Main Text).

**Table 1.** CD8^+^ T cell model parameters. Parameters and 95% confidence intervals obtained from fitting the CD8^+^ T cell model (Equation (1)-(6)) to viral titers and total CD8^+^ T cells (‘Total CD8’) or viral titers, CD25^–^CD43^+^CD8^+^ T cells, and CD43^–^CD127^+^CD103^+^CD69^+^CD8^+^ T cells (‘Specific CD8_E,M_ Phenotypes’) from mice infected with 75 TCID_50_ PR8. CD8_E_ and CD8_M_ denote effector (E) and memory ($E_{M}$) CD8^+^ T cells, respectively. The total number of CD8^+^ T cells is $\hat{E}=E+E_{M}+\hat{E_{0}}$ and is denoted by CD8.

Parameter	Description	Units	Total CD8		Specific CD8_E,M _phenotypes	
			Value	95% CI	Value	95% CI
β	Virus infectivity	${\mathrm{TCID}}_{50}^{-1}\,d^{-1}$	6.2 × 10^–5^	[5.3 × 10^–6^, 1.0 × 10^–4^]	3.7 × 10^–5^	[1.1 × 10^–5^, 9.4 × 10^–5^]
k	Eclipse phase transition	$d^{-1}$	4.0	[4.0, 6.0]	5.1	[4.0, 6.0]
p	Virus production	${\mathrm{TCID}}_{50}\,{\mathrm{cell}}^{-1}\,d^{-1}$	1.0	[5.8 × 10^–1^ × 1.1 × 10^2^]	1.5	[0.73, 13.6]
c	Virus clearance	$d^{-1}$	9.4	[5.6, 9.5 × 10^2^]	12.1	[5.8, 17.5]
δ	Infected cell clearance	$d^{-1}$	2.4 × 10^–1^	[1.0 × 10^–1^, 6.6 × 10^–1^]	3.0 × 10^–1^	[1.9 × 10^–1^, 5.9 × 10^–1^]
$\delta_{E}$	Infected cell clearance by CD8_E_	$\mathrm{cells}\,{\mathrm{CD8}}_{E}^{-1}\,d^{-1}$	1.9	[3.3. × 10^–1^, 2.0]	5.7	[1.7, 8.5]
$K_{\delta_{E}}$	Half-saturation constant	cells	4.3 × 10^2^	[1.0 × 10^2^, 2.9 × 10^5^]	1.3 × 10^2^	[1.0 × 10^1^, 8.6 × 10^2^]
ξ	CD8_E_ infiltration	$CD8_{E}^{2}\;{cell}^{-1}\;d^{-1}$	2.6 × 10^4^	[1.3 × 10^2^, 8.7 × 10^4^]	2.9 × 10^3^	[8.0 × 10^2^, 1.7 × 10^4^]
$K_{E}$	Half-saturation constant	${\mathrm{CD8}}_{E}$	8.1 × 10^5^	[1.0 × 10^3^, 1.0 × 10^6^]	2.2 × 10^6^	[1.1 × 10^6^, 8.3 × 10^6^]
η	CD8_E_ expansion	${\mathrm{cell}}^{-1}\,d^{-1}$	2.5 × 10^–7^	[1.6 × 10^–8^, 6.7 × 10^–7^]	3.5 × 10^–7^	[2.3 × 10^–7^, 5.2 × 10^–7^]
$\tau_{E}$	Delay in CD8_E_ expansion	d	3.6	[2.1, 5.9]	3.3	[2.6, 3.8]
$d_{E}$	CD8_E_ clearance	$d^{-1}$	1.0	[5.1 × 10^–2^, 2.0]	1.1	[3.3 × 10^–1^, 2.5]
ζ	CD8_M_ generation	$CD8_{M}\;CD8_{E}^{-1}\;d^{-1}$	2.2 × 10^–1^	[1.0 × 10^–2^, 9.4 × 10^–1^]	3.2 × 10^–2^	[1.0 × 10^–2^, 2.2 × 10^–1^]
$\tau_{M}$	Delay in CD8_M_ generation	d	3.5	[3.0, 4.0]	3.3	[2.1, 5.3]
${\hat{E}}_{0}$	Baseline CD8 or CD8_E_	CD8 or ${\mathrm{CD8}}_{E}$	4.2 × 10^5^	[3.3. × 10^5^, 5.3 × 10^5^]	6.0 × 10^2^	[3.1 × 10^2^, 1.8 × 10^2^]
${\hat{E}}_{M\,0}$	Baseline CD8_M_	${\mathrm{CD8}}_{M}$	-	-	6.5 × 10^2^	[3.2 × 10^2^, 1.5 × 10^3^]
T⁢(0)	Initial uninfected cells	cells	1 × 10^7^	-	1 × 10^7^	-
$I_{1}\,\left(0\right)$	Initial infected cells	cells	75	-	75	-
$I_{2}\,\left(0\right)$	Initial infected cells	cells	0	-	0	-
V⁢(0)	Initial virus	${\mathrm{TCID}}_{50}$	0	-	0	-
E⁢(0)	Initial CD8_E_	${\mathrm{CD8}}_{E}$	0	-	0	-
$E_{M}\,\left(0\right)$	Initial CD8_M_	${\mathrm{CD8}}_{M}$	0	-	0	-

Plotting the model predicted dynamics for the lung-specific CD8^+^ T cells (${\mathrm{CD8}}_{L}=E+E_{M}$) illustrated the accuracy of the model in predicting their dynamics within the lung parenchyma without fitting to these data (Figure 1C). One benefit of using the total CD8^+^ T cells is that the model automatically deduces the dynamics of effector-mediated killing and memory generation without needing to specify which phenotypes might be involved in these processes as they may be dynamically changing. However, the rates of expansion, contraction, and infected cell clearance may be different if only certain phenotypes are engaged. Thus, we examined whether the model could fit the dynamics of the predominant effector (CD25^–^CD43^+^) and memory (CD43^–^CD127^+^CD103^+^CD69^+^) phenotypes. Re-fitting the model to these data suggested that no changes to model formulation were needed and there were only small alterations to select CD8-specific parameter values (Figure 1D; Table 1).

Plotting the model ensembles revealed a correlation between the two infected cell clearance parameters (δ and $\delta_{E}$; Figure 2B), which represent the efficacy of the non-specific immune response and the CD8^+^ T cell response, respectively. Performing a sensitivity analysis showed that the viral load dynamics do not change substantially when these parameters are increased or decreased (Appendix 2—figure 1) . However, decreasing the rate of non-specific infected cell clearance (i.e., lower δ) resulted in a significant increase in the number of CD8_E_ due to the small increase in the number of infected cells (Figure 1). Even with a larger CD8_E_ population, recovery was delayed by only ~0.1 d. Given the correlation between δ and $\delta_{E}$ (Figure 2B), a more efficient CD8_E_ response (i.e., higher $\delta_{E}$) may be able to overcome this short delay in resolution. The lack of sensitivity to changes in the infected cell clearance parameters is in contrast to the DD model, where the viral dynamics were most sensitive to perturbations in $\delta_{d}$ (Appendix 2—figure 1; Smith et al., 2018), which encompasses multiple processes. With CD8^+^ T cells explicitly included in the model, the infection duration was most sensitive to changes in the rate of CD8_E_ expansion (η) (Figure 1, Appendix 1—figure 1; discussed in more detail below).

Examining the parameter ensembles and sensitivity analysis also yielded insight into how other model parameters affect the CD8^+^ T cell response. The rates of CD8_E_ expansion (η) and clearance ($d_{E}$) were slightly correlated, indicating a balance between these two processes (Figure 2A). This correlation and the sensitivity of η produced model dynamics that were also sensitive to changes in the CD8_E_ clearance rate ($d_{E}$) (Appendix 3—figure 2). As expected, the rates of CD8_M_ generation (ζ) and CD8_E_ clearance ($d_{E}$) were correlated (Figure 2A). It has been estimated that approximately 5–10% of effector CD8^+^ T cells survive to become a long-lasting memory population (Kaech et al., 2002). Despite the inability to distinguish between CD8_E_ and CD8_M_ in the total CD8^+^ T cell data, the model predicts that 17% of CD8_E_ transitioned to a memory class by 15 d pi. When considering only the CD25^–^CD43^+/–^ effector and memory phenotypes, the model estimates this value to be ~7%. Additional insight into the regulation of the CD8^+^ T cell response, results from the model fitting, and a comparison of the DD model and the CD8^+^ T cell model are included in Appendix 2.

#### Density-dependent infected cell clearance

**Figure 3.**
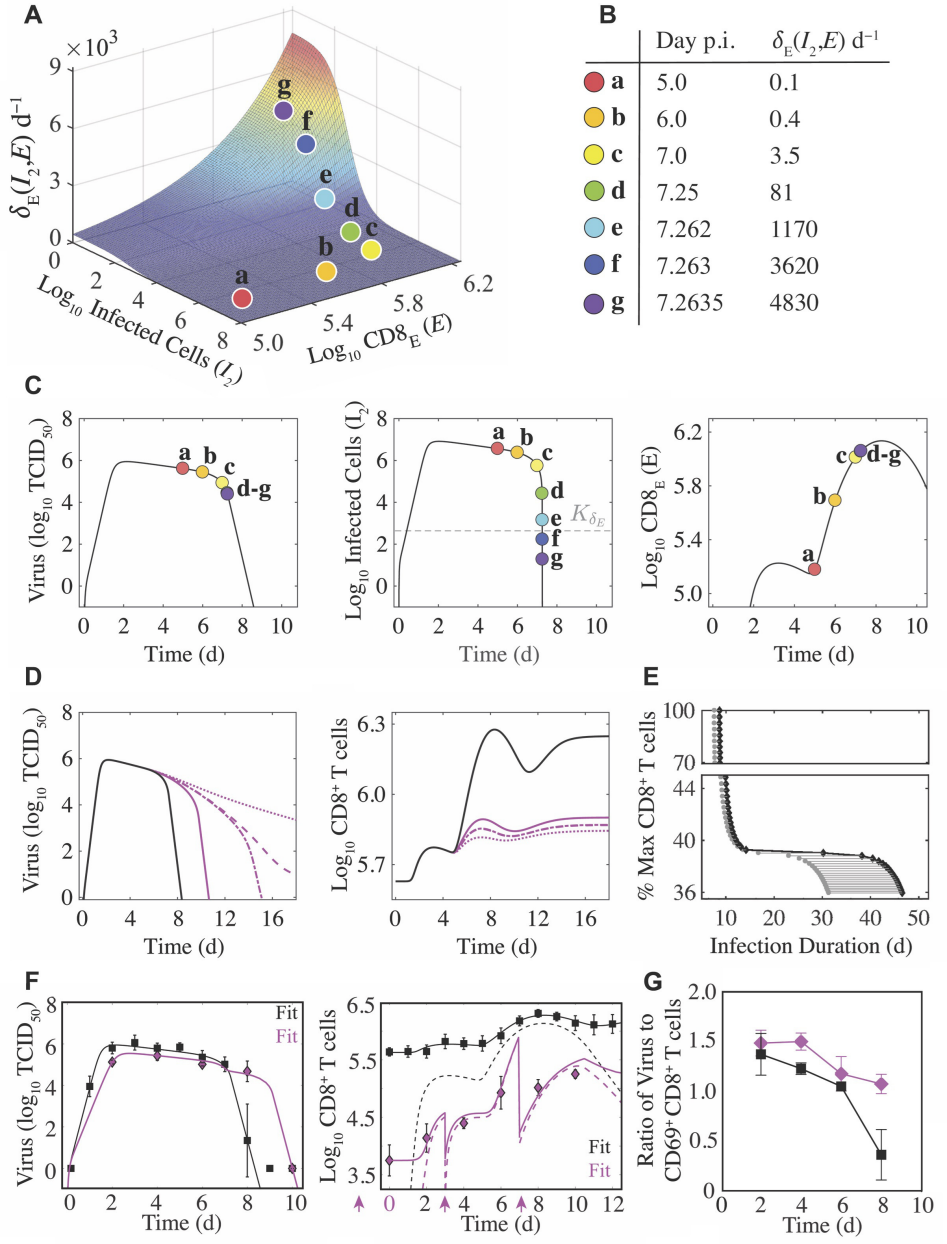
Density-dependent infected cell clearance by CD8^+^ T cells and their impact on recovery time. (**A**) The rate of CD8_E_-mediated infected cell clearance ($\delta_{E}\,\left(I_{2},E\right)=\delta_{E}\,E/\left(K_{\delta_{E}}+I_{2}\right)$) plotted as a function of infected cells ($I_{2}$) and effector CD8^+^ T cells (E; CD8_E_). The colored markers (denoted a–g) indicate the infected cell clearance rate that corresponds to different time points during the infection for the best-fit solution. (**B**) Values of $\delta_{E}\,\left(I_{2},E\right)$ for the indicated time points associated with the markers a–g. (**C**) Corresponding locations of the various $\delta_{E}\,\left(I_{2},E\right)$ values (markers a–g) on the best-fit solution of the CD8^+^ T cell model for virus (V), infected cells ($I_{2}$), and CD8_E_ (E). (**D**) Solutions of the CD8^+^ T cell model (Equation (1)-(6)) for virus (V) and total CD8^+^ T cells ($\hat{E}$) using the best-fit parameters (black line) and when varying the CD8_E_ expansion rate (η; magenta lines) to illustrate how different total CD8^+^ T cell magnitudes alter infection duration. The magenta lines are solutions from when the percent ${\hat{E}}_{\max}$ relative to ${\hat{E}}_{\max}$ from the best-fit solution was 42% (solid line), 39.2% (dash-dotted line), 39.1% (dashed line), or 37% (dotted line). (**E**) The time at which infected cells reach the half-saturation constant ($I_{2}=K_{\delta_{E}}$; gray circles) and the infection duration (time where $\log_{10}V=0$; black diamonds) are shown for the various CD8^+^ T cell magnitudes. The gray line between these points is the time required to eliminate $K_{\delta_{E}}$ infected cells and achieve complete resolution of the infection ($\log_{10}V=0$). (**F**) Fit of the CD8^+^ T cell model (Equation (1)-(6)) to viral loads and CD8^+^ T cells (magenta diamonds) following depletion at −2, 0, 3, and 7 d pi (magenta arrows). The best model (Supplementary file 2) resulted in fewer target cells ($T_{0}$), a lower CD8_E_ influx (ξ), and a higher CD8_E_ expansion rate (η). All other parameters were fixed to the best-fit value in Table 1. The solid lines are $\hat{E}=E+E_{M}+{\hat{E}}_{0}$ and the dashed lines are E for the cases where CD8^+^ T cells were depleted (magenta) and where they were not depleted (black). (**G**) Comparison of the log_10_ ratio of virus to CD69^+^CD8^+^ T cells with and without CD8^+^ T cell depletion (magenta and black, respectively). All data are shown as mean ± standard deviation.

Given the accuracy of the model, we next sought to gain further insight into the nonlinear dynamics of CD8^+^ T cell-mediated infected cell clearance. Plotting the clearance rate ($\delta_{E}\,\left(I_{2},E\right)=\delta_{E}\,E/\left(K_{\delta_{E}}+I_{2}\right)$) as a function of infected cells ($I_{2}$) and CD8_E_ (E) (Figure 3) confirmed that there is minimal contribution from CD8_E_-mediated clearance to viral load kinetics or infected cell kinetics prior to 7 d pi (Figure 3A–C, markers a–b). At the initiation of the second decay phase (7 d pi), the clearance rate is $\delta_{E}\,\left(I_{2},E\right)=3.5/d$ (Figure 3A–B, marker c). As the infected cell density declines toward the half-saturation constant ($K_{\delta_{E}}=4.3\times 10^{2}\,\mathrm{cells}$), the clearance rate increases rapidly to a maximum of 4830/d (Figure 3A–C, markers d–g). The model predicts that there are 6 × 10^5^ infected cells remaining at 7 d pi, which can be eliminated by CD8_E_ in 6.7 h.

To explore how recovery time is altered by varying CD8_E_ levels, we examined the resulting dynamics from increasing or decreasing the rate of CD8_E_ expansion (η). When η was increased by 50%, the CD8_E_ population increased by a substantial 670% (Appendix 3—figure 1). However, this was insufficient to significantly shorten the infection (8.4 d versus 7.8 d). The infection duration could be reduced if CD8_E_ expansion began earlier (i.e., smaller $\tau_{E}$; Appendix 3—figure 2). Although recovery is not significantly expedited by a larger CD8_E_ population, our model predicted that the infection would be dramatically prolonged if these cells are sufficiently depleted (Figure 3D–E and Figure 1). This *in silico* analysis revealed a bifurcation in recovery time, such that the infection is either resolved within ∼15 d pi or may last up to ∼45 d if CD8_E_ are below a critical magnitude required to resolve the infection (Figure 3D–E). The critical number of total CD8^+^ T cells needed for successful viral clearance was ${\hat{E}}_{max}^{crit}=7.4\times 10^{5}$ CD8, which was 39.2% of the maximum number of CD8^+^ T cells obtained from the best-fit solution (i.e., ${\hat{E}}_{max}=1.9\times 10^{6}$ CD8). This corresponds to 17% of CD8_E_ (i.e., $E_{max}^{crit}=2.3\times 10^{5}$ CD8_E_; Figure 3D–E). The model analysis indicated that decreasing the total number of CD8^+^ T cells by as little as 0.1% from this critical level (i.e., 39.2% to 39.1%) lengthened the infection from 15 d pi to 25 d pi (Figure 3D).

#### Dynamical changes during CD8^+^ T cell depletion

To further test our model formulation and identify how viral load kinetics are altered with dynamically changing CD8^+^ T cells, we infected groups of mice with 75 TCID_50_ and depleted these cells at −2 d, 0 d, 3 d, and 7 d pi with an anti-CD8α antibody (clone 2.43; Figure 3F). CD8^+^ T cells were reduced with over 99% efficiency and only 1.3% remained 2 d after depletion in the absence of infection (i.e., at 0 d pi; Figure 3F). CD8^+^ T cells remained >1 log_10_ lower throughout the infection. Unexpectedly, the corresponding viral loads were significantly lower at 2 d pi (p=0.02) and consistently lower at other time points early in the infection (4 d pi (p=0.1) and 6 d pi (p=0.2)). As expected and predicted by our mathematical model, viral loads were significantly higher at 8 d pi (4.68 log_10_ TCID_50_ compared to 1.42 log_10_ TCID_50_; p=0.01). By 10 d pi, a sufficient number of CD8^+^ T cells were present and all animals had cleared the infection (Figure 3F). Interestingly, the number of CD8^+^ T cells at 10 d pi was only slightly higher than at 8 d pi (5.25 log_10_ versus 5.02 log_10_; p=0.064) further highlighting the density-dependent dynamics described above.

Given that viral loads were lower at early time points and that the anti-CD8α antibody is known to cause concentration-dependent changes in CD8_E_ differentiation, activation, and efficacy (Cross et al., 2019) in addition to resulting in death of the cells that would initiate activation of and removal by other immune cells, we did not expect our model to match the data without modulation of parameter values. However, we did expect that no changes to the model formulation would be required. In total, we tested >30 ‘models’ where 1–4 parameters were altered and found one model that was significantly better according to the AIC (Supplementary file 1). In that model, the initial number of target cells ($T_{0}$) was 2.5x lower (~4 × 10^6^ versus 1 × 10^7^ cells), the rate of initial CD8_E_ influx (ξ) was 2x lower (1.3 × 10^4^ versus $2.6\times 10^{4}\;{CD8}_{E}^{2}\;{cell}^{-1}\;d^{-1}$), and the rate of CD8_E_ expansion (η) was 4x higher (1 × 10^–6^ versus $2.5\times 10^{-7}\;{cell}^{-1}\;d^{-1}$). The second best model had a lower cost but was penalized by an additional parameter. That model suggested similar results but replaced the effect on $T_{0}$ with a combination of a lower virus production rate (p) and higher non-specific infected cell clearance rate (δ). Both models resulted in fewer infected cells and, thus, have approximately equivalent interpretations.

To further examine these findings, we plotted the ratio of virus to activated (CD69^+^) CD8^+^ T cells (Figure 3G). At 2 d pi, the ratio was similar for the depleted and mock-treated groups (p=0.41) suggesting that the depletion-induced reduction in virus was proportional to the reduction in activated CD8^+^ T cells. However, at 4 d pi, the ratio in the depleted groups was significantly higher (p=0.0028) for otherwise similar levels of virus. This signifies that there were disproportionately low numbers of CD8^+^ T cells and, thus, reduced influx (i.e., lower ξ). By 6 d pi, the ratios were again similar (p=0.25) implying a higher expansion of these cells (i.e., higher η). Investigation into the predicted target cell reduction is included below.

### Modeling lung injury dynamics

**Figure 4.**
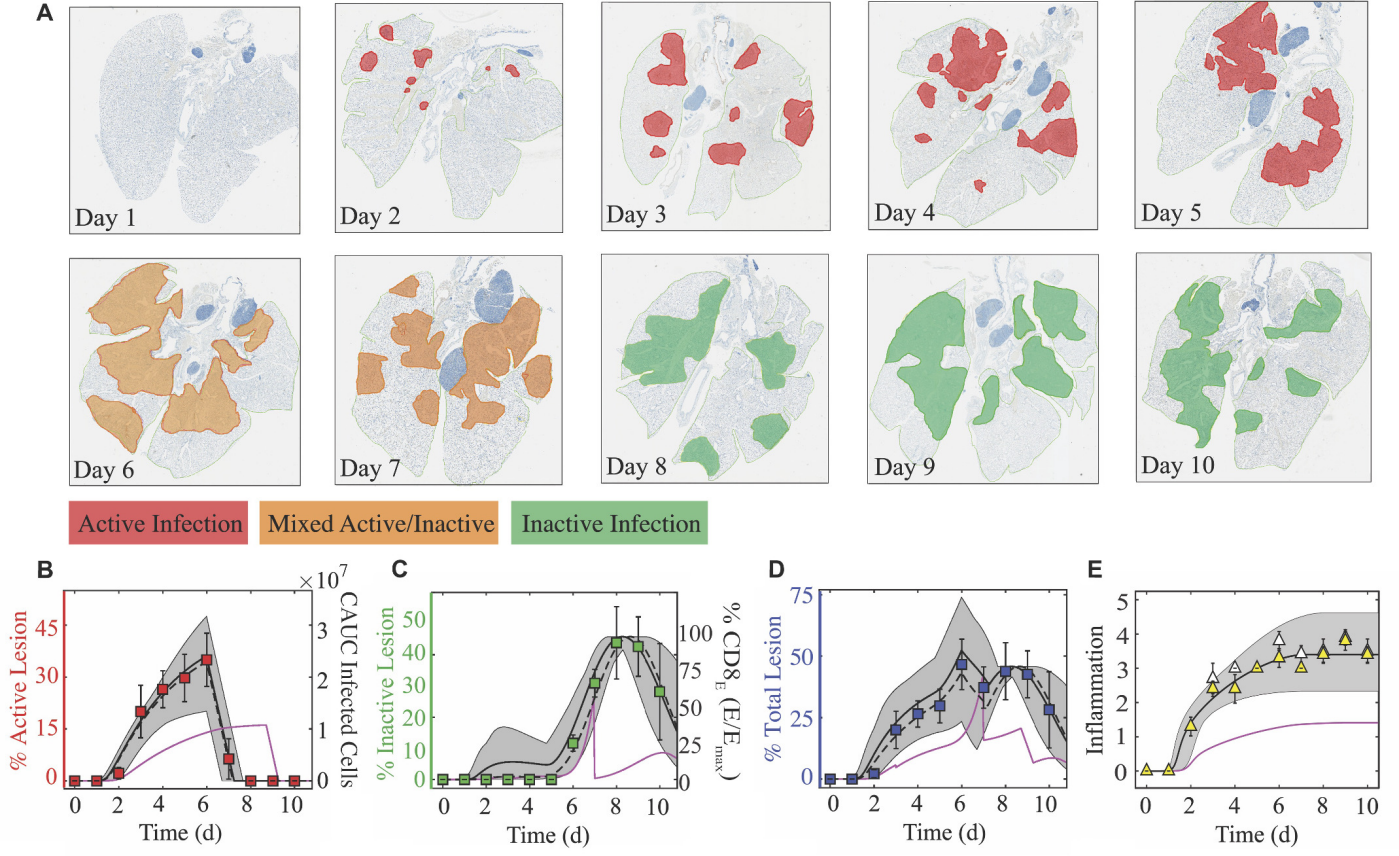
Histomorphometry and inflammation of the IAV-infected lung. (**A**) Whole lung sections with histomorphometry showing the areas of influenza NP-positive ‘active’ lesions (red), previously infected ‘inactive’ lesions with minimal antigen-positive debris (green), or mixed active and inactive regions (orange) throughout the infection. Representative images from each group are shown. (**B**) Percent active lesion (red squares) plotted together with the cumulative area under the curve (CAUC) of the predicted infected cell dynamics ($I_{2}$) obtained from fitting the CD8^+^ T cell model. The linear decline in the active lesion (−28.7%/d; see Figure 4—figure supplement 1) was used to estimate the decline after 6 d pi. (**C**) Percent inactive lesion (green squares) plotted together with the percent maximum CD8_E_ ($E/E_{\max}$) obtained from fitting the CD8^+^ T cell model. (**D**) The total lesion (blue squares) is the addition of the active and inactive lesions. To include all measurements on the same scale, the CAUC of $I_{2}$ was multiplied by a scaling factor of 14.2% per 1 × 10^7^ cells, and the percent maximum CD8_E_ was multiplied by a scaling factor of 0.46%. (**E**) Fit of Equation (7) to the alveolar (white triangles) and interstitial (yellow triangles) inflammation scores. The solid black, dashed black, and solid magenta lines are the curves generated using the best-fit parameters obtained from fitting the model to the total CD8^+^ T cells, the CD25^–^CD43^+^ and CD43^–^CD127^+^CD103^+^CD69^+^ CD8^+^ T cells, and the total CD8^+^ T cells during CD8 depletion, respectively. The gray shading are the curves generated using the 95% CI parameters from fitting the model to the total CD8^+^ T cells. All data are shown as mean ± standard deviation.

To investigate the dynamics of infected cells and their clearance by CD8^+^ T cells, we quantified these cells and the progression and resolution of lung injury using serial whole lung histomorphometry (Figure 4A). Antigen-positive areas of the lung (‘active’ lesions) were first detectable at 2 d pi (Figure 4A–B), which coincides with the peak in viral loads. The infected area continued to increase in a nonlinear manner until 6 d pi, whereas viral loads remained high until 7 d pi (Figure 1B). At this time, resolution of the infection began and the infected area declined at a rate of ∼28.7%/d between 6–7 d pi (Figure 4—figure supplement 1). Few to no infected cells were present at 8 d pi (Figure 4A). Correspondingly, virus was undetectable in most animals by 8 d pi (Figure 1B). Because the percent active lesion is a reflection of the influenza-positive cells, we examined whether the CD8^+^ T cell model accurately predicted these dynamics. In the model, the accumulated infection is defined by the cumulative area under the curve (CAUC) of the productively infected cells ($I_{2}$). Plotting the percent active lesion against the CAUC of $I_{2}$ shows that the model accurately reflects the cumulative infected cell dynamics and, thus, the infection progression within the lung (Figure 4B). Plotting the CAUC of $I_{2}$ for all parameter sets in the 95% CIs and from fitting the model to specific phenotypes further illustrates the accuracy by showing that the heterogeneity in the histomorphometry data is captured (Figure 4B), which is larger than the heterogeneity in viral loads (Figure 1B). Plotting the predicted active lesion dynamics for the model parameterized to the data where CD8^+^ T cells were depleted suggested that there was a ∼22% reduction in the active lesion (Figure 4B).

**Figure 4—figure supplement 1.**
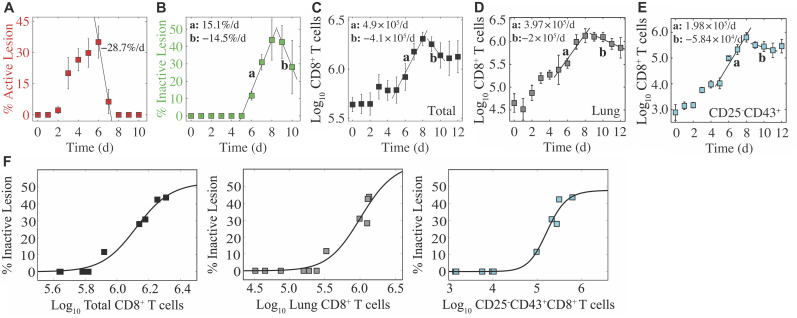
Regression analysis of lung injury dynamics and CD8^+^ T cells. (**A**) Percent active lesion area decreases by 28.7%/d from 6 -7 d pi. (**B**) Percent inactive lesion area increases by 15.1%/d from 5 -8 d pi, and decreases by 14.5%/d from 9-10 d pi. (**C**) Total CD8^+^ T cells increase at a rate of 4.9 × 10^5^ cells/d from 5-8 d pi, and decrease at a rate of 4.1 × 10^5^ cells/d from 9-10 d pi. (**D**) Lung CD8^+^ T cells increase at a rate of 3.97 × 10^5^ cells/d from 5-8 d pi, and decrease at a rate of 2 × 10^5^ cells/d from 9-12 d pi. (**E**) CD25^+^CD43^+^CD8^+^ T cells increase at a rate of 1.98 × 10^5^ cells/d from 5-8 d pi, and decrease at a rate of 5.84 × 10^4^ cells/d from 9-11 d pi. (**F**) Fit of a Hill function ($L_{I}=c_{m\,a\,x}\,E^{n}/\left(K_{L}^{n}+E^{n}\right)$) to the total CD8^+^ T cells (black; $c_{max}=52.88$, $K_{L}=6.12$, n=56.32), lung CD8^+^ T cells (gray; $c_{max}=64.65$, $K_{L}=6.02$, n=25.16), and CD25^+^CD43^+^CD8^+^ T cells (cyan; $c_{max}=48.04$, $K_{L}=5.23$, n=24.03).

**Figure 4—figure supplement 2.**
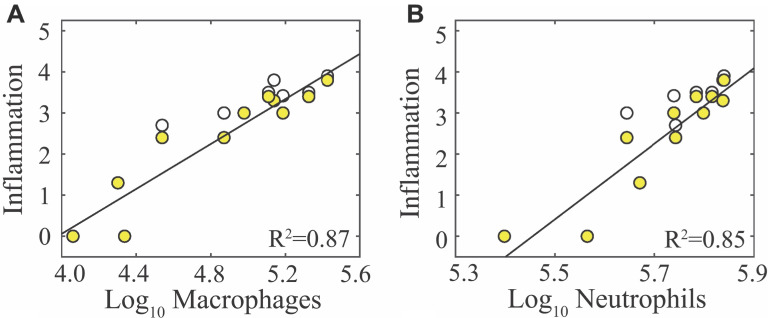
Correlation of inflammation with macrophages and neutrophils. Regression analysis of alveolar inflammation (white) and interstitial inflammation (yellow) with (**A**) log_10_ inflammatory macrophages (F480^hi^CD11c^hi^CD11b^+^) and (**B**) log_10_ neutrophils (Ly6G^hi^).

Antigen-negative, previously-infected or damaged areas of the lung (‘inactive’ lesions) are evident beginning at 5 d pi (Figure 4A,C). This resolution of the infection continued from 5-8 d pi, causing a 15.1%/d increase in the area of inactive lesions (Figure 4—figure supplement 1). Following this, healing of the injured lung is apparent as the inactive lesioned area declines (−14.5%/d from 9–10 d pi; Figure 4C and Figure 4—figure supplement 1). These dynamics generally parallel the CD8^+^ T cell dynamics but are nonlinearly correlated (Figure 4—figure supplement 1). Fitting a line to the CD8^+^ T cell data from 5-8 d pi indicated that the influx rate of all phenotypes is 4.94 × 10^5^ cells/d, of lung-specific phenotypes is 3.97 × 10^5^ cells/d, and of the CD25^–^CD43^+^ effector phenotype is 1.98 × 10^5^ cells/d (Figure 4—figure supplement 1). Thus, the model estimates that, on average, every 100,000 total, lung-specific, or CD25^–^CD43^+^ CD8^+^ T cells clear ~3.1%, ~3.8%, or ~7.6% of the infected areas within the lung, respectively. During the CD8^+^ T cell contraction phase, a similar linear regression analysis suggested that these cells decline at rates of ~ 4.13 × 10^5^ CD8/d (total), ~ 2.35 × 10^4^ CD8/d (lung-specific), and ~ 3.83 × 10^4^ CD8/d (CD25^–^CD43^+^) (Figure 4—figure supplement 1). Similar to the relation discussed above, the dynamics of the damaged areas of the lung corresponded to the dynamics of the percent maximum CD8_E_ (i.e., $E/E_{max}$) in the model (Figure 4C). Our model suggested a ∼37% reduction in the inactive lesion during CD8^+^ T cell depletion (Figure 4C). Adding the predicted dynamics for the active and inactive lesions agrees with the dynamics of the total lesion, but is slightly underestimated when using the model fit to CD25^–^CD43^+^ CD8^+^ T cells (Figure 4D). In addition, the predicted total lesion was reduced for the case where CD8^+^ T cells were depleted (Figure 4D).

### Modeling lung inflammation dynamics

In addition to measuring virus-induced lung damage, lung inflammation was scored (Figure 4E). Both alveolar and interstitial inflammation begin to increase at 2 d pi with the sharpest increase between 1–3 d pi. Inflammation continues to increase until 6 d pi with a maximum score of 3.5–4.0 out of 5. Resolution of inflammation was not evident during the time course of our data, which concluded at 10 d pi. This is in contrast to the lung damage inflicted by the virus, which begins declining at 8 d pi and shows that ∼15% of the damage was repaired by 10 d pi. Inflammation was linearly correlated to inflammatory macrophages and to neutrophils (Figure 4—figure supplement 2), which were excluded from the model. However, the knowledge that the model’s predicted infected cell dynamics are accurate and that these cells can produce cytokines and chemokines that attract cells like macrophages and neutrophils suggested that our model could be used to estimate inflammation. To do this, we fit Equation (7) to the inflammation scores while keeping all other parameters fixed to their best-fit values (Table 1). The results suggested that the equation captured the inflammation dynamics, and that the contribution from the initial infected cell class ($I_{1}$) was α1=4.27⁢per⁢ 107⁢cells/d and the contribution from the productively infected cells ($I_{2}$) was α2=0.87⁢per⁢ 107⁢cells/d. For the case where CD8^+^ T cells were depleted, Equation (7) estimated the inflammation scores to be reduced to ∼1.5 (Figure 4E).

### Weight loss relates nonlinearly to lung injury and inflammation

**Figure 5.**
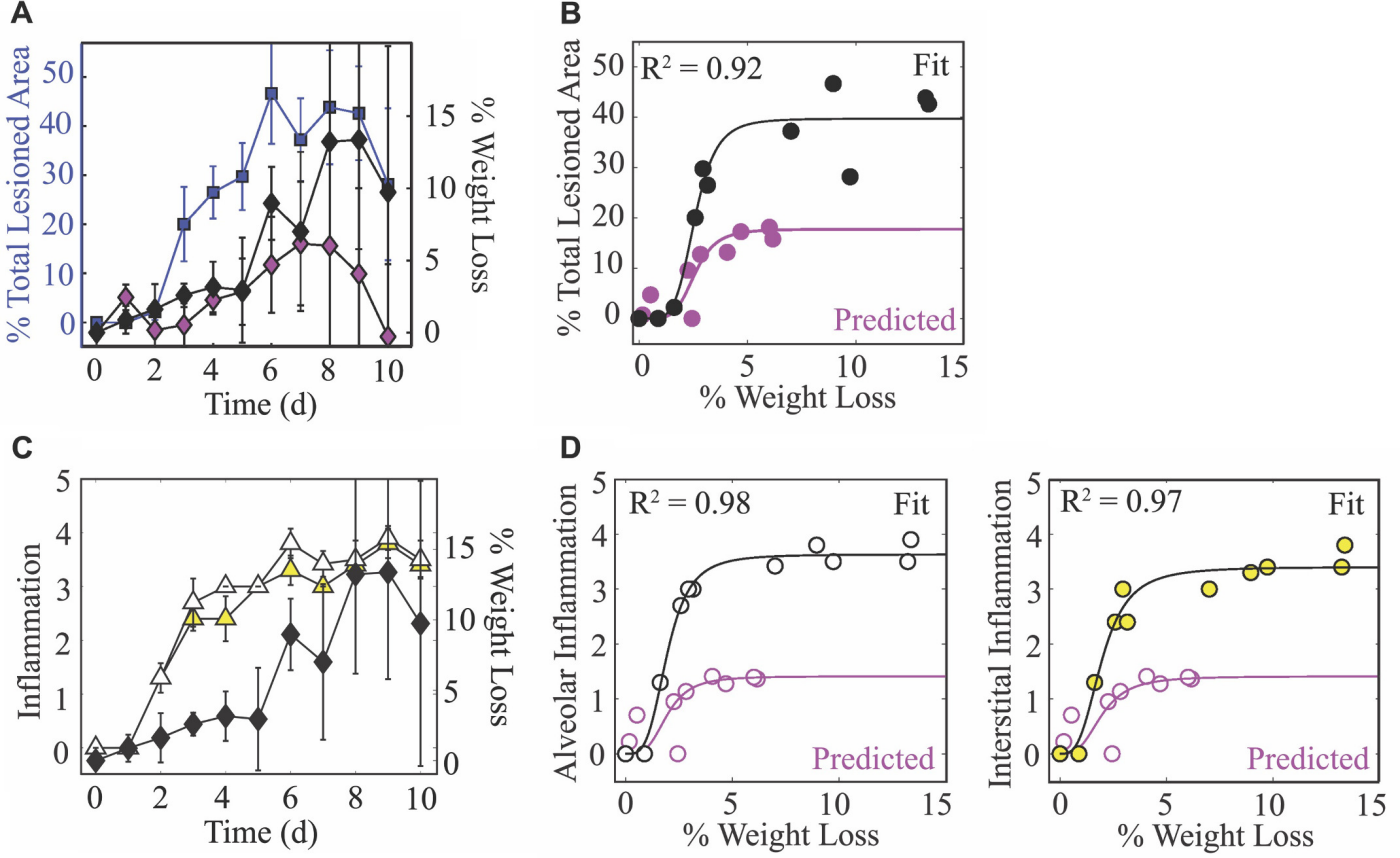
Weight loss dynamics and its relation to lung injury and inflammation. (**A**) The percent total (active and inactive) lesion (blue squares) plotted together with the percent weight loss (black diamonds) to illustrate their similar dynamics. (**B**) Fit of a saturating function ($L\,\left(W\right)=l_{\max}\,W^{n}/\left(K_{w}^{n}+W^{n}\right)$; black line) to the mean percent total lesioned area (L) and mean weight loss (W) for all time points (black circles). The best-fit parameters were $l_{\max}=39.7\%$ total lesioned area, $K_{w}=2.6\%$ weight loss, and n=5.2. The magenta circles are the predicted percent total lesioned area (Figure 4D) with the corresponding weight loss during CD8 depletion, and the magenta line is the prediction using the best-fit parameters ($K_{w}=2.6\%$ weight loss and n=5.2) together with the maximum predicted percent total lesion ($l_{\max}=17.73\%$ total lesioned area). (**C**) The alveolar (white triangles) and interstitial (yellow triangles) inflammation plotted together with the percent weight loss (black diamonds). (**D**) Fit of a saturating function (black line) to the mean alveolar (white circles) and interstitial (yellow circles) inflammation scores and mean weight loss for all time points. The best-fit parameters for alveolar inflammation (white circles; black line) were $l_{a_{max}}=3.63$ score, $K_{a_{w}}=1.95\%$ weight loss, and $n_{a}=3.65$, and for interstitial inflammation were $l_{i_{max}}=3.40$ score, $K_{i_{w}}=1.96\%$ weight loss, and $n_{i}=3.15$. The magenta circles are the predicted percent inflammation score (Figure 4E) with the corresponding weight loss during CD8 depletion, and the magenta line is the prediction using the best-fit parameters ($K_{a_{w}}=1.95$% weight loss, $n_{a}=3.65$; $K_{i_{w}}=1.96\%$ weight loss, $n_{i}=3.15$) together with the maximum predicted inflammation ($l_{max}=1.41$ score).

To monitor disease progression, weight loss was measured daily throughout the course of infection (Figure 5). During the first 5 d pi, animals gradually lost ∼4% of their initial weight. This was followed by a sharper drop (8%) at 6 d pi. Animal weights increased slightly at 7 d pi (∼6%) before reaching peak weight loss (10–14%) at 8 d pi. Following virus resolution, the animals’ weights began to restore as the inactive lesions resolved (9–10 d pi; Figure 5A). Weight loss was reduced during CD8^+^ T cell depletion (Figure 5A). Plotting weight loss against the percent total (active and inactive) lesioned area of the lung shows the similarity in their dynamics (Figure 5A) and revealed a nonlinear relation (Figure 5B). To further quantify their relationship, we fit the saturating function $L(W)=l_{max}W^{n}/(W^{n}+K_{w}^{n})$ to these data, where L is the percent total lesioned area, W is percent weight loss, *l*_*max*_ is the maximum rate of the interaction, $K_{w}$ is the half-saturation constant, and n is the Hill coefficient. This function provided a close fit to the data ($R^{2}=0.92$; black line in Figure 5B) with best-fit parameters $l_{max}=39.7\%$ total lesioned area, $K_{w}=2.58\%$ weight loss, and n=5.24. Plotting the estimated percent total lesion during CD8^+^ T cell depletion (Figure 4D) together with the measured weight loss (Figure 5A) also supported the nonlinear relation. Using the same best-fit parameters ($K_{w}=2.58\%$ weight loss and n=5.24) together with the predicted maximum percent total lesion ($l_{max}=17.73\%$ total lesioned area) showed the accuracy of this function (magenta line in Figure 5D). Weight loss was also nonlinearly correlated to the alveolar and interstitial inflammation scores (Figure 5C), although the relation was slightly different compared to that for the lung injury data. Fitting the same function to these data independently for alveolar and interstitial inflammation also provided a close fit ($R^{2}=0.98$ and $R^{2}=0.97$; black lines in Figure 5D). Best-fit parameters for alveolar inflammation were $l_{a_{\max}}=3.63$ score, $K_{a_{w}}=1.95\%$ weight loss, and $n_{a}=3.65$. Best-fit parameters for interstitial inflammation were $l_{i_{\max}}=3.40$ score, $K_{i_{w}}=1.96\%$ weight loss, and $n_{i}=3.15$. For the CD8-depleted case, plotting the estimated inflammation (Figure 4E) together with the measured weight loss (Figure 5A) again supported the nonlinear relation. In addition, using the same best-fit parameters ($K_{a_{w}}=1.95\%$ weight loss, $n_{a}=3.65$; $K_{i_{w}}=1.96\%$ weight loss, $n_{i}=3.15$) together with the predicted maximum inflammation score ($l_{i_{\max}}=1.41$ score) showed the accuracy (magenta line in Figure 5D).

## Discussion

**Figure 6.**
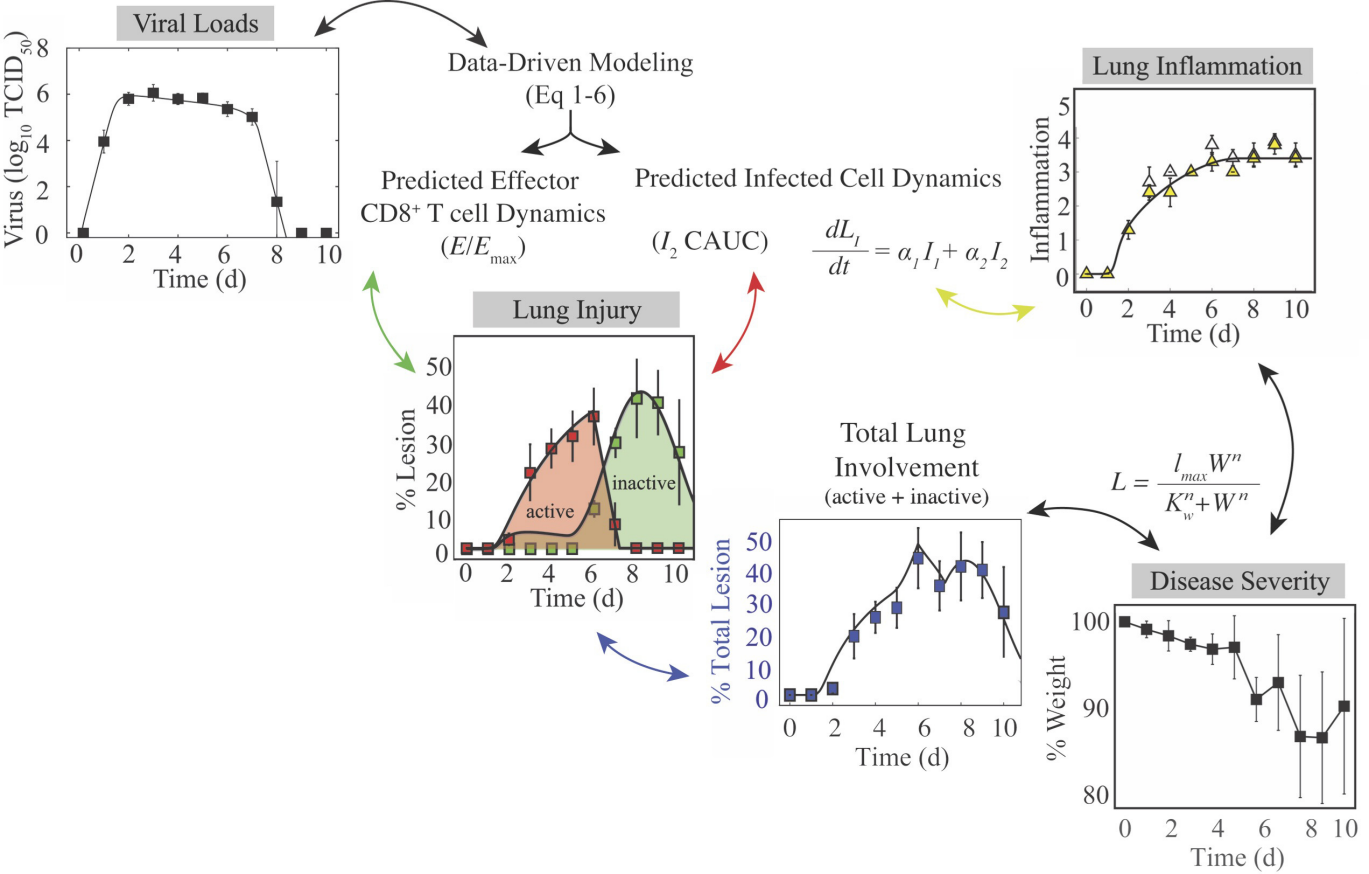
Summary of the connections between the kinetics of virus, infected cells, CD8^+^ T cells, lung injury, lung inflammation, and disease severity. Summary of the relations between the dynamics of virus, infected cells, CD8^+^ T cells, lung lesions, lung inflammation, and weight loss established by our analysis. Given that viral loads and weight loss are the most easily measured variables, our mathematical model (Equation (1)-(6)) could be used to estimate the kinetics of infected cells, CD8^+^ T cells, and inflammation. The cumulative area under the curve (CAUC) of the productively infected cell dynamics ($I_{2}$) yields an estimate of the percent lung infected (active lesion) while the predicted relative CD8_E_ dynamics ($E/E_{m\,a\,x}$) yields an estimate of the percent lung resolved (inactive lesion). The total amount of lung involved (% lung infected and % lung resolved) or the inflammation scores can then be used to estimate weight loss through the functions $L_{L}\,\left(W\right)$ and $L_{I}\,\left(W\right)$. These connections could be reversed and weight loss used to estimate viral load kinetics.

Influenza A virus infections pose a significant threat to human health, and it is crucial to understand and have tools that can predict how the virus spreads within the lower respiratory tract, how specific immune responses contribute to infection control, and how these relate to disease progression. Although it has been difficult to directly relate these features and obtain high quality data from the lower respiratory tract in humans, we circumvented the challenge by pairing comprehensive experimental data with robust mathematical models and analyses. Our iterative model-driven experimental approach (Smith, 2018a; Smith, 2018b) revealed important dynamic relations between virus, infected cells, immune cells, lung damage, inflammation, and disease severity (summarized in Figure 6). Identifying these nonlinear connections allows for more accurate interpretations of viral infection data and significant improvement in our ability to predict disease severity, the likelihood of complications, and therapeutic efficacy.

Our histomorphometric data and analyses provided a robust quantification of the extent of influenza virus infection within the lung and the dynamics of lung injury and inflammation. The images look strikingly similar to CT scans and postmortem histopathology from infected patients, which also show extensive bilateral damage and cytopathic effects in epithelial cells (Kohr et al., 2010; Li et al., 2011; Ajlan et al., 2009; Soto-Abraham et al., 2009; Gill et al., 2010; Li et al., 2012; Shieh et al., 2010). In addition, our results agree with weekly CT imaging of hospitalized patients infected with the 2009 H1N1 influenza virus showing that the damage worsens during the first week of illness and that cell proliferation begins in the second week (Li et al., 2012). Further investigating the utility of CT imaging could enhance the translatability of our findings. The model’s 95% CI predictions of the lesioned area nicely captured the heterogeneity in the data (Figure 4), which is remarkable given the minimal variability in the virus and CD8^+^ T cell data and model fit (Figure 1). The differential heterogeneity suggests that small changes to lung viral loads can induce significant changes in disease severity. Indeed, this has been observed in both humans and animals when antivirals are administered (Toniolo Neto, 2014; Baloxavir Marboxil Investigators Group et al., 2018; Treanor et al., 2000; Moscona, 2005; Gubareva et al., 2000; Koshimichi et al., 2018) or when host responses are experimentally modulated (Channappanavar et al., 2016; Szretter et al., 2009; Iwasaki and Nozima, 1977). It was also observed in our CD8 depletion data, where our model predicted a significant reduction in the lesioned area (Figure 4) while viral loads were only slightly lower (Figure 3). This may indicate that viral loads are generally not a reliable measure of infection severity, and the knowledge should aid future studies seeking to estimate therapeutic efficacy or the effects of immune modulation. There was a slight over-estimation of the inactive lesion early in the infection (1–5 d pi, dark gray shading; Figure 4) when using the fit to the total CD8^+^ T cells, which was abrogated when using the CD25^-^CD43^+^CD8^+^ T cells. However, these cells underestimated the total lesion at 6–7 d pi, suggesting contributions from other CD8^+^ phenotypes. In general, modeling different CD8^+^ T cell phenotypes may help clarify their *in vivo* functions and better define the rates of CD8-mediated infected cell clearance. Although our results suggest that this is not necessarily required, they may be more critical to consider when predicting the response of preexisting and potentially cross-reactive immunity particularly given that memory resident T cells are only beginning to be understood (Zens et al., 2016; Slütter et al., 2017; Van Braeckel-Budimir et al., 2018; Wakim et al., 2015; Uddbäck et al., 2021).

The new knowledge about the extent of infection within the lung also uncovered the nonlinear connection between disease severity and various measures of lung pathology (Figure 5). This discovery is significant because it suggests there is a unique link to the extent of both lung injury and inflammation, which are often used interchangeably yet have distinct dynamics (Figure 4) and correlate to different immune responses (Figure 4—figure supplements 1–2). In the same vein, both measurements can be estimated using the dynamics of infected cells (Figure 4) but with independent mathematical relations (CAUC of $I_{2}$ versus Equation (7)). Our CD8 depletion data (Figure 3) and other experimental studies using histomorphometry (Marathe et al., 2016) corroborate a relationship between weight loss and lung pathology during IAV infection. For example, animals treated with antivirals in various conditions (single or combination therapy and in immunocompetent or immunosuppressed hosts) demonstrated that, although viral loads are not always significantly reduced, the antiviral-induced reductions in weight loss were paired with decreased infected areas of the lung (Marathe et al., 2016). Further examining lung injury and inflammation kinetics and their connection to weight loss in various experimental infection settings (e.g., different ages or sex, under therapy, other strains or viruses, etc.) and deciphering how innate and humoral responses contribute to these measurements should improve our predictive capabilities. In addition, the nonlinearity of the connections warrants further investigation as it could indicate cooperativity of multiple mechanisms. Studies such as these may be particularly helpful in understanding the exacerbated morbidity and mortality in elderly individuals, where there are lower viral loads, increased weight loss, symptoms, and/or mortality within animal models (Toapanta and Ross, 2009; Smith et al., 2019). Because murine models are imperfect systems with important immunologic and physiologic distinctions from humans (Mestas and Hughes, 2004), uncovering how our results translate to human infection with novel or circulating influenza viruses and identifying the corresponding, tractable human symptom that could be a robust predictor of disease would be ideal. However, this could be arduous due to inherent human and viral heterogeneity and contributions from co-morbidities and complex immunologic histories.

Despite their limitations, animal models are used to make viral and immunological discoveries and to develop and test therapeutics and vaccines (reviewed in Thangavel and Bouvier, 2014; Margine and Krammer, 2014; Smith and McCullers, 2014), and this work demonstrates a significant potential for the easily obtained weight loss data to be used, analyzed, and interpreted within both modeling and experimental studies. In our data and others’ animal data, there is a spike in weight loss, symptom score, and/or inflammation at ∼6 d pi (Music et al., 2014; Kugel et al., 2009; Lu et al., 2018; Huang et al., 2017; Figure 5). Our data suggests that this may be due to CD8_E_ activity while the infection continues to spread. The increase in animal weights starting at 7 d pi is concurrent with the decline in infected lesions (Figure 5). Interestingly, there was no corresponding decrease in inflammation, and the spike was not apparent in the CD8 depletion data where severity was reduced. The subsequent increase in weight loss following infection resolution (8–9 d pi; Figure 4C) could be attributed to CD8^+^ T cell-mediated pathology and/or to ongoing inflammation. The tighter correlation to inflammation might suggest the latter; however, some human and animal studies indicate that large numbers of CD8^+^ T cells pose a risk of acute lung tissue injury (Duan and Thomas, 2016; Moskophidis and Kioussis, 1998; Mauad et al., 2010; Rygiel et al., 2009; La Gruta et al., 2007). According to our model predictions, excessive CD8^+^ T cell numbers may augment disease progression yet do not improve recovery time (Appendix 3—figure 2). Instead, an earlier onset of CD8_E_ proliferation (i.e., smaller $\tau_{E}$) would be required to significantly shorten the infection (Appendix 3—figure 2). This aligns with evidence that hosts with adaptive immune responses primed by vaccine or prior infection recover more rapidly (Duan et al., 2015; Weinfurter et al., 2011). While higher CD8^+^ T cell numbers have little impact on viral kinetics, our model and data agree with clinical and experimental studies from a wide range of host species that impaired CD8^+^ T cell responses can prolong an IAV infection (Wang et al., 2015; Miao et al., 2010; McMichael et al., 1983; Yap and Ada, 1978; Wells et al., 1981; Bender et al., 1992). In some scenarios, such as in immunocompromised hosts, virus can persist for up to several weeks if CD8^+^ T cell-mediated clearance is unsuccessful (Figure 3; Wang et al., 2015; Eichelberger et al., 1991; Hou et al., 1992; Xue et al., 2017; Memoli et al., 2014). The bifurcation in recovery time revealed by the model suggests that this may occur when the number of lung-specific CD8^+^ T cells are less than 2.31 × 10^5^ cells (Figure 3D–E). Our CD8 depletion data show that the precise number will be dependent on other infection variables. Minimally, we would expect this number to vary depending on parameters like the dose, rate of virus replication, degree of prior immunity, and/or the infected cell number and lifespan, which has been noted in another modeling study that detailed similar bifurcating behavior (Yates et al., 2011). Although some previously published models also suggested delayed resolution with depleted CD8^+^ T cell responses (Dobrovolny et al., 2013; Miao et al., 2010; Cao et al., 2016; Lee et al., 2009), this bifurcation has not been observed and their estimated delays in recovery do not amount to the long-lasting infections in immunodeficient patients (Wang et al., 2015; Eichelberger et al., 1991; Hou et al., 1992). Our model’s ability to capture the dynamics when CD8^+^ T cells are depleted is encouraging, and the data are an important reminder that experimental modulation of cell populations (e.g., through depletion or genetic knockouts) is complicated and that the data from such systems should be interpreted cautiously. It also brings into question studies that have used these types of data without validated mathematical models or quantification of other immunological variables.

Our data and analyses provided strong support that the infected cell density impacts the rate at which they are cleared by effector CD8^+^ T cells (CD8_E_) (Figures 3–4, Figure 4—figure supplement 1). Although CD8^+^ T cell dynamics can be somewhat replicated when assuming the density dependence lies within their expansion, that assumption could not recover the viral load dynamics (Appendix 1—figure 1). Discriminating between these mechanisms is difficult *in vivo,* but the ability of the model in Equation (1)-(6) to capture the entire time course of CD8_E_ dynamics in multiple scenarios is compelling. Regardless of the mechanism, we first detected this density-dependence in a model that excluded specific immune responses (see Appendix 2) (Smith et al., 2018). Simple models like that one are useful to capture nonlinearities, but they cannot distinguish between different mechanisms.

Several factors may contribute to the density-dependent change in the rate of CD8_E_-mediated clearance. One possibility is that the slowed rate of clearance at high infected cell densities is due to a ‘handling time’ effect, which represents the time required for an immune cell to remove an infected cell (e.g., as in Smith et al., 2018; Li and Handel, 2014; Le et al., 2014; Graw and Regoes, 2009; Gadhamsetty et al., 2014; Pilyugin and Antia, 2000; Smith et al., 2011b). When CD8_E_ interact with infected cells, a complex is formed for ∼20–40 min (Mempel et al., 2006; Deguine et al., 2010; Zagury et al., 1975; Yannelli et al., 1986; Perelson and Bell, 1982). Because CD8_E_ could not interact with other infected cells during this time, the global rate of infected cell clearance would be lowest when infected cells outnumber CD8_E_. In addition, contact by a single CD8_E_ may be insufficient to remove an infected cell (Halle et al., 2016). Infected cell clearance is more frequently observed after interactions with multiple CD8_E_, with an average of 3.9 contact events either serially or simultaneously (Halle et al., 2016). Thus, the high density of infected cells early in the infection reduces the probability that a single virus-infected cell would be targeted a sufficient number of times to induce cell death. However, as CD8_E_ accumulate and the density of infected cells decreases (Figure 3A), the probability of simultaneous interactions will increase. These interactions may also be influenced by CD8_E_ movement, where their velocity slows at the height of infected cell clearance (6–8 d pi) (Lambert Emo et al., 2016; Matheu et al., 2013). This should reduce the time required to remove an infected cell and, thus, result in a higher efficiency. Moreover, it is possible that spatial limitations also contribute, such that a high infected cell density may hinder CD8^+^ T cells from reaching infected cells on the interior of the infected tissue. Crowding of immune cells at the periphery of infected lesions has been observed in other infections (e.g., in tuberculosis granulomas [Ulrichs et al., 2004; Russell et al., 2009]) and has been suggested in agent-based models of influenza virus infection (Levin et al., 2016).

In addition to illuminating the connections between viral kinetics and pathology, the histomorphometric data validated the model’s infected cell dynamics (Figure 4B). The dynamics of susceptible and infected cells throughout the infection and the accuracy of the target cell limited approximation used within influenza viral kinetic models have been questioned for several years (Smith and Perelson, 2011; Smith, 2018b; Beauchemin and Handel, 2011; Ahmed et al., 2017; Gallagher et al., 2018; Boianelli et al., 2015; Handel et al., 2018). The ability of the model to accurately predict the histomorphometry and CD8^+^ T cell depletion data corroborates the use of this approximation, which assumes a limited number of available target cells and describes their decline by infection only. Although the slowing of viral loads beginning at 2 d pi could be due to a variety of innate immune-mediated mechanisms (e.g., macrophages, neutrophils, type I interferons, etc.), adding more complex dynamics to our model was unnecessary to describe the data. Further, the model and data agree that there are few infected cells during the time when viral loads are growing most rapidly (0–2 d pi; Figures 1B and 4B). We previously used this information to derive approximations for the model and gain deeper insight into how each parameter influences the kinetics (Smith et al., 2010), which has helped numerous studies interpret their results (Miao et al., 2010; Smith et al., 2011a; Smith, 2018b; Holder and Beauchemin, 2011). The data also supports the model’s hypothesis that that there is minimal clearance of infected cells prior to CD8_E_ expansion (Figure 4). The knowledge of the model’s accuracy and of the spatiality in the lung should aid investigation into the mechanisms that limit virus growth during the early stages of the infection.

Employing targeted model-driven experimental designs to examine and validate theoretical predictions like the ones presented here is pivotal to elucidating the mechanisms of infection spread and clearance (Smith, 2018b). Examining other infections (e.g., coronaviruses), modifications to the dynamics (e.g., lethal doses), and the connection between lung measurements and those more easily acquired from upper respiratory tract will help refine the dynamical links between virus, host immune responses, and disease severity and identify their generalizability. Determining the factors that influence disease severity is vital to understanding the disproportionate mortality in at-risk populations (e.g., elderly) and to improving therapeutic design. This is particularly important because current antivirals alleviate symptoms but do not always effectively lower viral loads (Toniolo Neto, 2014; Baloxavir Marboxil Investigators Group et al., 2018; Treanor et al., 2000; Moscona, 2005; Gubareva et al., 2000; Koshimichi et al., 2018). The predictive capabilities of validated models like the one here should prove useful in forecasting infection dynamics for a variety of scenarios. These tools and analyses provide a more meaningful interpretation of infection data, new ways to interpret weight loss data in animal models, and a deeper understanding of the progression and resolution of the disease, which will undoubtedly aid our ability to effectively combat influenza.

## Materials and methods

### Ethics statement

All experimental procedures were performed under protocols O2A-020 or 17–096 approved by the Animal Care and Use Committees at St. Jude Children’s Research Hospital (SJCRH) or the University of Tennessee Health Science Center (UTHSC), respectively, under relevant institutional and American Veterinary Medical Association (AVMA) guidelines. All experimental procedures were performed in a biosafety level two facility that is accredited by the American Association for Laboratory Animal Science (AALAS).

### Mice

Adult (6-week-old) female BALB/cJ mice were obtained from Jackson Laboratories (Bar Harbor, ME) or Charles River Laboratories (Wilmington, Massachusetts). Mice were housed in groups of five mice in high-temperature 31.2 cm × 23.5 cm × 15.2 cm polycarbonate cages with isolator lids (SJCRH) or in 38.2 cm × 19.4 cm × 13.0 cm solid-bottom polysulfone individually ventilated cages (UTHSC). Rooms used for housing mice were maintained on a 12:12 hr light:dark cycle at 22 ± 2°C with 50% humidity in the biosafety level two facility at SJCRH (Memphis, TN) or UTHSC (Memphis, TN). Prior to inclusion in the experiments, mice were allowed at least 7 days to acclimate to the animal facility such that they were 7 weeks old at the time of infection. Laboratory Autoclavable Rodent Diet (PMI Nutrition International, St. Louis, MO; SJCRH) or Teklad LM-485 Mouse/Rat Sterilizable Diet (Envigo, Indianapolis, IN; UTHSC) and autoclaved water were available *ad libitum*. All experiments were performed under an approved protocol and in accordance with the guidelines set forth by the Animal Care and Use Committee at SJCRH or UTHSC.

### Infectious agents

All experiments were done using the mouse adapted influenza A/Puerto Rico/8/34 (H1N1) (PR8).

### Infection experiments

The viral infectious dose (TCID_50_) was determined by interpolation using the method of Reed and Muench, 1938 using serial dilutions of virus on Madin-Darby canine kidney (MDCK) cells. Mice were intranasally inoculated with 75 TCID_50_ PR8 diluted in 100 μl of sterile PBS. In a subset of animals, CD8^+^ T cells were depleted by IP injection at days −2, 0, 3, and 7 pi with 100 μg of the rat anti-CD8α antibody (clone 2.43) that was purified from ATCC hybridoma (per manufacturer instructions) in 250 μl of PBS. Depletion efficiency was confirmed in the lung and spleen as described below. Mice were weighed at the onset of infection and each subsequent day to monitor illness and mortality. Mice were euthanized if they became moribund or lost 30% of their starting body weight. For viral load and total CD8^+^ T cell quantification, experiments were repeated three times and in each facility to ensure reproducibility and two complete experiments (ten animals per time point) were used for these studies. For all other experiments, the data was compared to prior results in addition to being repeated at select time points to ensure reproducibility, and one complete experiment (five animals per time point) was used. For pathology scoring and histomorphometry quantitation, five animals per time point were used. Power was calculated using G*Power.

### Lung harvesting for viral and cellular dynamics

For total CD8^+^ T cell quantification, mice were euthanized by CO_2_ asphyxiation. To distinguish blood-borne CD8^+^ T cells from those in the lung parenchyma, deeply anesthetized mice (5% isoflurane) were retro-orbitally injected with 3 μg of anti-CD45 antibody (PerCP, clone 30-F11, Biolegend) 3 min prior to euthanasia (Anderson et al., 2012; Keating et al., 2018). Mice were then euthanized by 33% isoflurane inhalation and their lungs perfused with 10 ml PBS prior to removal. For all experiments, lungs were then aseptically harvested, washed three times in PBS, and placed in 500 μl sterile PBS. Whole lungs were digested with collagenase (1 mg/ml, Sigma C0130), and physically homogenized by syringe plunger against a 40 μm cell strainer. Cell suspensions were centrifuged at 4°C, 500xg for 7 min. The supernatants were used to determine the viral titers (TCID_50_) by serial dilutions on MDCK monolayers. Following red blood cell lysis, cells were washed in MACS buffer (PBS, 0.1M EDTA, 0.01M HEPES, 5 mM EDTA and 5% heat-inactivated FBS). Cells were then counted with trypan blue exclusion using a Cell Countess System (Invitrogen, Grand Island, NY) and prepared for flow cytometric analysis as indicated below.

### Lung titers

For each animal, viral titers were obtained using serial dilutions on MDCK monolayers and normalized to the total volume of lung homogenate supernatant. The resulting viral loads are shown in Figure 1B, provided in Source data 1, and were previously published and utilized for calibration of the density-dependent model (see Appendix 2; Smith et al., 2018).

### Flow cytometric analysis

Flow cytometry (LSRII, BD Biosciences, San Jose, CA (SJCRH) or ZE5 Cell Analyzer, Bio-Rad, Hercules, CA (UTHSC)) was performed on the cell pellets after incubation with 200 μl of a 1:2 dilution of Fc block (human-γ globulin) on ice for 30 min, followed by viability (Biolegend, Zombie Violet Fixable Viability Kit) and surface marker staining with anti-mouse antibodies. For total CD8^+^ T cell, macrophage, and neutrophil quantification, we used antibodies CD3e (BV786, clone 145–2 C11, Biolegend), CD4 (PE-Cy5, clone RM4-5, BD Biosciences), CD8α (BV605, clone 53–6.7, BD Biosciences), Ly6C (APC, clone HK1.4, eBioscience), F4/80 (PE, clone BM8, eBioscience), CD11c (eFluor450, clone N418, eBioscience), CD11b (Alexa700, clone M1/70, BD Biosciences), MHC-II (FITC, clone M5/114.15.2, Invitrogen), CD49b (APCe780, clone DX5, eBioscience), and Ly6G (PerCp-Cy5.5, clone 1A8, Biolegend). The data were analyzed using FlowJo 10.4.2 (Tree Star, Ashland, OR) where viable cells were gated from a forward scatter/side scatter plot and singlet inclusion. Following neutrophil (Ly6G^hi^) and subsequent macrophage (CD11c^hi^F4/80^hi^) exclusion, CD8^+^ T cells were gated as CD3e^+^DX5^–^CD4^–^CD8^+^.

To distinguish blood borne CD8^+^ T cells from those in the lung parenchyma, we used antibodies CD3e (BV786, clone 145–2 C11, Biolegend), CD4 (FITC, clone RM4-5, Biolegend), CD8α (BV605, clone 53–6.7, BD Biosciences), B220 (APCe780, clone RA3-6B2, eBioscience), CD49b (APCe780, clone DX5, eBioscience), CD62L (PE-Cy7, clone MEL-14, Biolegend), CD69 (PE, clone H1.2F3, Biolegend), CD44 (PE-Dazzle594, clone IM7, Biolegend), CD25 (Alexa700, clone PC61, Biolegend), CD43 (APC, clone 1B11, Biolegend), CD127 (PE-Cy5, clone A7R34, Biolegend), and CD103 (BV711, clone 2E7, Biolegend) or CD314 (NKG2D) (BV711, clone CX5, BD Bioscience). The data were analyzed using FlowJo 10.6.2 (Tree Star, Ashland, OR) where viable cells were gated from a forward scatter/side scatter plot, singlet inclusion, and viability dye exclusion. Blood borne CD8^+^ T cells were gated as CD45^+^ and those in the lung parenchyma as CD45^-^. The total in each sub-population was gated as CD3e^+^B220^–^DX5^–^CD4^–^CD8^+^. IAV-specific CD8^+^ T cells were then gated as CD25^+^CD43^+^ (recently activated), CD25^–^CD43^+^ (effector) (Keating et al., 2018), and CD43^–^CD127^+^CD103^+^CD69^+^ (long-lived memory). Expression of CD44, CD69, CD62L, and NKG2D were also assessed to ensure appropriate classification.

For all experiments, the absolute numbers of CD8^+^ T cells were calculated based on viable events analyzed by flow cytometry as related to the total number of viable cells per sample. The data are provided in Source data 1. We use the kinetics of the total number of CD8^+^ T cells (non-perfused lung; ‘total’), the total number of CD8^+^ T cells in the lung parenchyma (perfused lung; ‘lung-specific’), and virus-specific CD8^+^ T cell phenotypes in the lung parenchyma as defined by surface staining (described above). We chose this approach because the use of tetramers yields epitope-specific cell dynamics that vary in time and magnitude (e.g., as in Toapanta and Ross, 2009; Keating et al., 2018) and would complicate the model and potentially skew the results. In addition, tetramer staining is not available for all epitopes in BALB/cJ mice and they may underestimate the dynamics of IAV-specific cells (Keating et al., 2018). The gating strategies are shown in Figure 1—figure supplement 2.

### Lung immunohistopathologic and immunohistochemical (IHC) evaluation

The lungs from IAV-infected mice were fixed via intratracheal infusion and then immersion in 10% buffered formalin solution. Tissues were paraffin embedded, sectioned, and stained for influenza virus using a primary goat polyclonal antibody (US Biological, Swampscott, MA) against influenza A, USSR (H1N1) at 1:1000 and a secondary biotinylated donkey anti-goat antibody (sc-2042; Santa Cruz Biotechnology, Santa Cruz, CA) at 1:200 on tissue sections subjected to antigen retrieval for 30 min at 98°C. The extent of virus spread was quantified by capturing digital images of whole-lung sections (two dimensional) stained for viral antigen using an Aperio ScanScope XT Slide Scanner (Aperio Technologies, Vista, CA) then manually outlining defined fields. Alveolar areas containing virus antigen-positive pneumocytes were highlighted in red (defined as ‘active’ infection), whereas lesioned areas containing minimal or no virus antigen-positive debris were highlighted in green (defined as ‘inactive’ infection). Lesions containing a mix of virus antigen-positive and antigen-negative pneumocytes were highlighted in orange (defined as ‘mixed’ infection). The percentage of each defined lung field was calculated using the Aperio ImageScope software. Pulmonary lesions in HE-stained histologic sections were assigned scores based on their severity and extent. Representative images from each group and quantitative analyses (five animals/group) of viral spread and lung pathology during IAV infection are shown in Figure 4, and provided in Source data 1.

### CD8^+^ T cell model

To examine the contribution of CD8^+^ T cells to the biphasic viral load decay, we expanded the density-dependent (DD) model (see Appendix 2) to include two mechanisms of infected cell clearance (non-specific clearance (δ) and CD8^+^ T cell-mediated clearance ($\delta_{E}(I_{2},E)$)) and two CD8^+^ T cell populations: effector (E, denoted CD8_E_) and memory ($E_{M}$, denoted CD8_M_) CD8^+^ T cells. The model is given by Equation (1)-(6).

(1) $$\frac{d\,T}{d\,t}=-\beta \,T\,V$$

(2) $$\frac{d\,I_{1}}{d\,t}=\beta \,T\,V-k\,I_{1}$$

(3) $$\frac{d\,I_{2}}{d\,t}=k\,I_{1}-\delta \,I_{2}-\delta_{E}\,\left(I_{2},E\right)\,I_{2}$$

(4) $$\frac{d\,V}{d\,t}=p\,I_{2}-c\,V$$

(5) $$\frac{d\,E}{d\,t}=\xi \,\left(E\right)\,I_{2}+\eta \,E\,I_{2}\,\left(t-\tau_{E}\right)-d_{E}\,E$$

(6) $$\frac{d\,E_{M}}{d\,t}=\zeta \,E\,\left(t-\tau_{M}\right)$$

In this model, target cells become infected with virus at rate β⁢V per day. Once infected, cells enter an eclipse phase ($I_{1}$) before transitioning at rate k per day to a productively-infected state ($I_{2}$). These infected cells produce virus at rate p TCID_50_ per infected cell per day, and virus is cleared at rate c per day. Virus-producing infected cells ($I_{2}$) are cleared by non-specific mechanisms (e.g., apoptosis and/or innate immune responses) at a constant rate δ per day. Innate immune responses were excluded from the model because the viral load data is linear during the time where they act (2–7 d pi) and, thus, additional equations cannot improve the fit. Productively infected cells are cleared by CD8_E_ at rate $\delta_{E}\,\left(I_{2},E\right)=\delta_{E}\,E/\left(K_{\delta_{E}}+I_{2}\right)$ per day, where the rate of infected cell clearance is $\delta_{E}/K_{\delta_{E}}$ per CD8_E_ per day and $K_{\delta_{E}}$ is the half-saturation constant. The CD8_E_-mediated clearance rate ($\delta_{E}\,\left(I_{2},E\right)$) is dependent on the density of infected cells and is similar to the infected cell clearance term in the DD model (see Equation (A11); Smith et al., 2018). Similar density-dependent forms have also been used in models that describe the CD8^+^ T cell response to other virus infections (De Boer and Perelson, 1998; Li and Handel, 2014; Gadhamsetty et al., 2014). Models that exclude this density-dependence were examined, but these models resulted in a statistically poor fit to the data as defined by the Akaike Information Criteria (AIC) (Supplementary file 2). This is due in part to the increase of CD8^+^ T cells from 3-5 d pi (Figure 1). We did examine a model that excluded these dynamics and included CD8^+^ T cell expansion as a density-dependent function (e.g., $\eta \,E\,I_{2}\,\left(t-\tau_{E}\right)/\left(K_{I}+I_{2}\right)$) while keeping a linear rate of CD8-mediated infected cell clearance ($\delta_{E}\,E\,I_{2}$) (see Appendix 1). This model was adapted from other published models (Conway and Perelson, 2015; Bonhoeffer et al., 2000; Baral et al., 2019) and can produce similar dynamics from 6-10 d pi, but it was not statistically supported by our data (Supplementary file 1). We further tested the model in Baral et al., 2019, but this model could not fit our data and lacked statistical support (Supplementary file 1).

The model assumes that the initial CD8_E_ influx in the lung is proportional to infected cells at rate $\xi (E)=\xi /(K_{E}+E)$ CD8_E_ per cell per day, which is down-regulated by the CD8_E_ already present in the lung. The associated half-saturation constant is $K_{E}$. Similar terms for CD8_E_ regulation have been used in modeling HIV infections (De Boer and Perelson, 1998; Müller et al., 2001) and in models that examined CD8^+^ T cell proliferation mechanisms (De Boer and Perelson, 1995). We also examined whether their influx is proportional to infected cells at a delay (i.e., $\xi \,\left(E\right)\,I\,\left(t-\tau_{I}\right)$). While this modification better captured the initial increase in CD8^+^ T cells, the additional parameter was not supported. In our model, CD8_E_ expansion occurs at rate η per infected cell per day with time delay $\tau_{E}$. This term accounts for local CD8_E_ proliferation in the lung (McGill and Legge, 2009; Lawrence et al., 2005) and migration of CD8_E_ from secondary lymphoid organs (Zhang and Bevan, 2011; Bedoui and Gebhardt, 2011; Wu et al., 2011; Kang et al., 2011). The delay may signify the time it takes CD8_E_ to become activated by antigen presenting cells, differentiate, proliferate, and/or migrate to the infection site. The lung CD8_E_ population declines due to cell death and/or emigration at rate $d_{E}$ per day. These cells transition to CD8_M_ ($E_{M}$) at rate ζ CD8_M_ per CD8_E_ per day after $\tau_{M}$ days. The model schematic and fit to the viral load, total, effector, and memory CD8^+^ T cell data are in Figure 1. All code is provided in Source code 1.

### Inflammation model

To estimate the alveolar and interstitial inflammation without modeling other cell classes (e.g., macrophages and neutrophils), we assumed that inflammation in the lung ($L_{I}$) was proportional to the infected cells ($I_{1}$ and $I_{2}$) according to the equation,

(7) $$\frac{dL_{I}}{dt}=\alpha_{1}I_{1}+\alpha_{2}I_{2},$$

where $\alpha_{1,2}$ has units of score/cell/d and defines the inflammation score contribution from each infected cell class. A decay term was excluded because inflammation does not resolve on the timescale of our data.

### Parameter estimation

Given a parameter set θ, the cost C⁢(θ) was minimized across parameter ranges using an Adaptive Simulated Annealing (ASA) global optimization algorithm (Smith et al., 2018) to compare experimental and predicted values of virus (V; log_10_ TCID_50_/lung) and log_10_ total CD8^+^ T cells/lung ($\hat{E}=E+E_{M}+{\hat{E}}_{0}$, where ${\hat{E}}_{0}$ is the initial number of CD8^+^ T cells at 0 d pi), or log_10_ TCID_50_/lung virus (V), log_10_ effector CD8^+^ T cells/lung ($\hat{E}=E+{\hat{E}}_{0}$), and log_10_ memory CD8^+^ T cells/lung (${\hat{E}}_{M}=E_{M}+{\hat{E}}_{M_{0}}$). The cost function is defined by,

$$\begin{array}{l} C(\theta )=\sum_{i,j}(V(\theta ,t_{i})-v_{i,j}{)}^{2}+\sum_{i,j}({\hat{E}}_{M}(\theta ,t_{i})-m_{i,j}{)}^{2}+s_{E}[\sum_{i,j}(\hat{E}(\theta ,t_{i})-e_{i,j}{)}^{2}+\\ \;\;\;\;\;\;\;\sum_{i}\sqrt{\gamma_{i}}{\left(\frac{\hat{E}(\theta ,t_{i+1})-\hat{E}(\theta ,t_{i-1})}{t_{i+1}-t_{i-1}}-\frac{1}{\gamma_{i}}\sum_{j}\frac{e_{i+1,j}-e_{i-1,j}}{t_{i+1}-t_{i-1}}\right)}^{2}], \end{array}$$

where $\left(t_{i},v_{i,j}\right)$ is the viral load data, $\left(t_{i},e_{i,j}\right)$ is the total or effector CD8^+^ T cell data, $\left(t_{i},m_{i,j}\right)$ is the memory CD8^+^ T cell data, and $V\,\left(\theta ,t_{i}\right)$, $\hat{E}\,\left(\theta ,t_{i}\right)$, and $\hat{E_{M}}\,\left(\theta ,t_{i}\right)$ are the corresponding model predictions. Here, $s_{E}=\left(v_{\text{max}}-v_{\text{min}}\right)/\left(e_{\text{max}}-e_{\text{min}}\right)$ is a scaling factor, and $\gamma_{i}=J_{i+1}\,J_{i-1}$ where $J_{i}$ is the number of observations at time $t_{i}$. Errors of the log_10_ data were assumed to be normally distributed. To explore and visualize the regions of parameters consistent with the model, we fit Equation (1)-(6) to 2000 bootstrap replicates of the data. If the fit was within $\chi^{2}=0.05$ of the best-fit and the CD8 derivative was not a statistical outlier as determined by the function *isoutlier*, then the bootstrap was considered successful (Smith et al., 2011a; Smith et al., 2013; Smith et al., 2018). For each best-fit estimate, we provide 95% confidence intervals (CI) obtained from the bootstrap replicates (Table 1). Calculations were performed either in MATLAB using a custom built ASA algorithm (Smith et al., 2018) or in Python using the *simanneal* package (Perry, 2018) followed by a L-BFGS-B (Byrd et al., 1995; Zhu et al., 1997) deterministic minimization through SciPy’s *minimize* function. MATLAB *ode15s* and *dde23* or SciPy integrate.ode using *lsoda* and PyDDE (Cairns, 2008) were used as the ODE and DDE solvers.

Estimated parameters in the CD8^+^ T cell model included the rates of virus infection (β), virus production (p), virus clearance (c), eclipse phase transition (k), non-specific infected cell clearance (δ), CD8_E_-mediated infected cell clearance ($\delta_{E}$), half-saturation constants ($K_{\delta_{E}}$ and $K_{E}$), CD8_E_ infiltration (ξ), CD8_E_ expansion (η), delay in CD8_E_ expansion ($\tau_{E}$), CD8_E _clearance ($d_{E}$), CD8_M_ generation (ζ), delay in CD8_M_ generation ($\tau_{M}$), and the baseline number of CD8^+^ T cells (${\hat{E}}_{0}$, ${\hat{M}}_{0}$). Bounds were placed on the parameters to constrain them to physically realistic values. Because biological estimates are not available for all parameters, ranges were set reasonably large based on preliminary results and previous estimates (Smith et al., 2018). The rate of infection (β) was allowed to vary between $10^{-6}-10^{-1}\,{\mathrm{TCID}}_{50}^{-1}\,d^{-1}$, and the rate of virus production (p) between $10^{-1}-10^{3}\,{\mathrm{TCID}}_{50}\,{\mathrm{cell}}^{-1}\,d^{-1}$. Bounds for the virus clearance rate (c) were $1\,d^{-1}$ ($t_{1/2}=16.7\,h$) and $10^{3}\,d^{-1}$ ($t_{1/2}=1\,\min$). To ensure biological feasibility, the lower and upper bounds for the eclipse phase transition rate (k) were 4-6 d^-1^ as done previously (Smith et al., 2018).

The rate of non-specific infected cell clearance (δ) was given limits of 0.05–1 d^-1^. The CD8_E_-mediated infected cell clearance rate ($\delta_{E}$) varied between $0.01-2\,\mathrm{cells}\,{\mathrm{CD8}}_{E}^{-1}\,d^{-1}$, and the associated half-saturation constant ($K_{\delta_{E}}$) was bounded between 10^1^–10^6^ cells. The upper bound of $\delta_{E}$ was chosen to maintain the convergence of δ to nonzero values and consistency with prior results where δ approximates the slope of the first decay phase (Smith et al., 2010). Bounds for the rate of CD8_E_ infiltration (ξ) were $10^{2}-10^{6}\;{CD8}_{E}^{2}\;{cell}^{-1}\;d^{-1}$, and bounds for the half-saturation constant ($K_{E}$) were 10^3^-10^7^ CD8_E_. The CD8_E_ expansion rate (η) varied between 10^–8^–10^–6^ cell^–1^ d^–1^, and the delay in CD8_E_ expansion ($\tau_{E}$) between 2–6 d. The rate of CD8_E_ clearance ($d_{E}$) had limits of 0.05–2 d^–1^. The rate of CD8_M_ generation (ζ) varied between $0.01-1\;CD8_{M}\;CD8_{E}^{-1}\;d^{-1}$, and the delay in CD8_M_ generation ($\tau_{M}$) varied between 3–4 d (total CD8^+^ T cell fit) or 2–6 d pi (effector and memory CD8^+^ T cell fit). Larger bounds were examined for this parameter; however, the parameter was non-identifiable in the total CD8^+^ T cell fit and a small range was required for convergence. Bounds for the baseline number of CD8^+^ T cells (${\hat{E}}_{0}$, ${\hat{E}}_{M_{0}}$) were set to the upper and lower values of the data at 0 d pi ($3.0\times 10^{5}-5.3\times 10^{5}\,\mathrm{CD8}$ (total), $2.5\times 10^{2}-1.4\times 10^{3}\,{\mathrm{CD8}}_{E}$ (effector), $3.4\times 10^{2}-8.0\times 10^{2}\,{\mathrm{CD8}}_{M}$ (memory)).

The initial number of target cells (T⁢(0)) was set to 10^7^ cells (Smith et al., 2011a). The initial number of infected cells $I_{1}\,\left(0\right)$ was set to 75 cells to reflect an initial dose of 75 TCID_50_ (Smith et al., 2018). We previously found that estimating $I_{1}\,\left(0\right)$, fixing $V\,\left(0\right)=75\,{\mathrm{TCID}}_{50}$, or estimating V⁢(0) did not improve the fit and could not be statistically justified (Smith et al., 2018). The initial number of productively infected cells ($I_{2}\,\left(0\right)$), the initial free virus (V⁢(0)), and the initial number of CD8_E_ (E⁢(0)) and CD8_M_ ($E_{M}\,\left(0\right)$) were set to 0. All other parameter estimations were done as described in the text.

### Linear regression

The function *polyfit* in MATLAB was used to perform linear regression on the percent active lesioned area, the percent inactive lesioned area, and the CD8^+^ T cells during the expansion phase (5–8 d pi) and the contraction phase (9–10 d pi). Linear fits are shown in Figure 4—figure supplement 1.

### Cumulative area under the curve

The function *cumtrapz* in MATLAB was used to estimate the cumulative area under the curve (CAUC) of the infected cells ($I_{2}$) for the best-fit model solution.

## Figures and Tables

**Appendix 1—figure 1.**
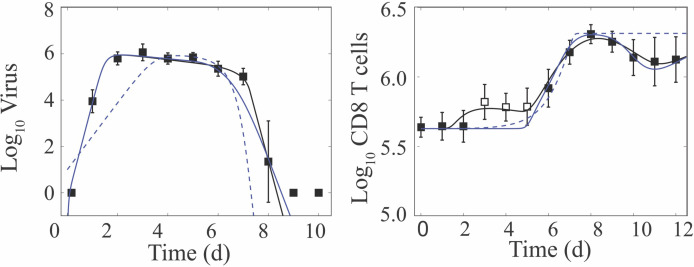
Fit of alternate CD8^+^ T cell models. Fit of the CD8^+^ T cell model (Equation (1)-(6); solid black line) compared to the fit of two alternate CD8^+^ T cell models (Equation (A1)-(A6) (solid blue line) and Equation (A7)-(A8) (dashed blue line)) to virus and CD8^+^ T cells (excluding 3–5 d pi; white squares) from the lungs of mice infected with 75 TCID_50_ PR8 (10 mice per time point). Resulting parameter values were $\delta_{Ea}=4.02\times 10^{-6}\;{CD8}_{E}^{-1}d^{-1}$, $\eta_{E\,a}=3.12\times 10^{5}\,{\mathrm{CD8}}_{E}/d$, and $K_{Ea}=9.53\times 10^{5}\;infected\;cells$, and $\kappa_{b}=3.33\,d^{-1}$, $I_{max}=1.0\;\times 10^{6}\;infected\;cells$, δE⁢b=1.53×10-5⁢CD8E-1⁢d-1, $\eta_{b}=1.49\,d^{-1}$, $K_{Eb}=9.5\;infected\;cells$, $d_{E\,b}=0.95\,d^{-1}$, and $K_{Db}=1.90\;\times 10^{6}\;infected\;cells$. All other parameters are in Table 1, and the AICs are in Supplementary file 1. Data are shown as mean ± standard deviation.

**Appendix 2—figure 1.**
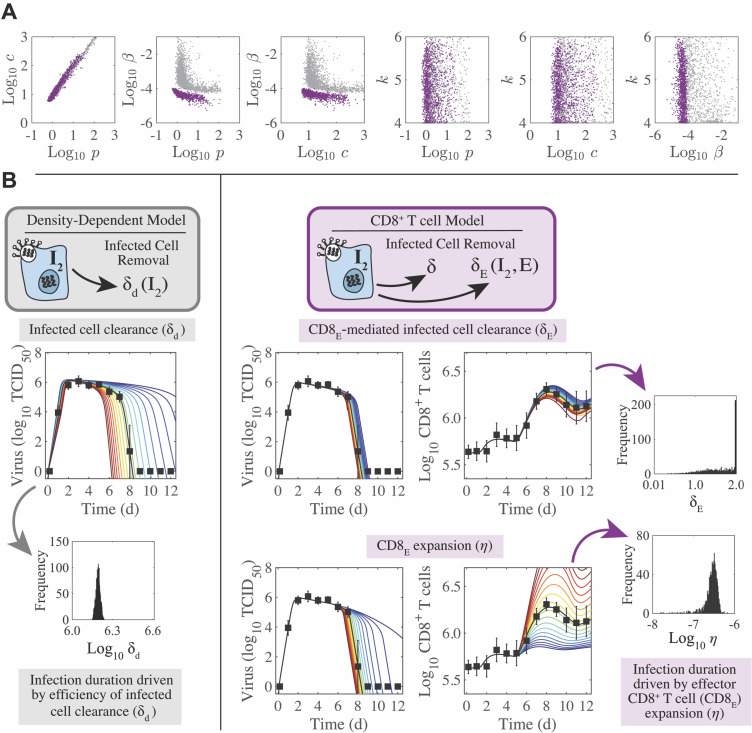
Parameter behavior of the density-dependent model and the CD8^+^ T cell model. (**A**) Comparison of parameters that were common between the density-dependent model (gray, Equation (A9)-(A12)) and the CD8^+^ T cell model (purple, Equation (1)-(6)). Correlations were evident between parameters relating to the rates of virus infectivity (β), virus production (p), and virus clearance (c). However, the strength of the correlation was significantly reduced in the CD8^+^ T cell model. The eclipse phase parameter (k) was not well-defined in either model. (**B**) In the density-dependent model (gray), the viral kinetics and the infection duration were sensitive to small changes in the infected cell clearance parameter ($\delta_{d}$). This parameter was well-defined with a narrow 95% CI. In the CD8^+^ T cell model (purple), changing the CD8_E_-mediated infected cell clearance parameter ($\delta_{E}$) had little impact on viral kinetics or CD8^+^ T cell kinetics. However, these kinetics were most sensitive to changes in the rate of CD8_E_ expansion (η), which was well-defined with a narrow 95% CI.

**Appendix 3—figure 1.**
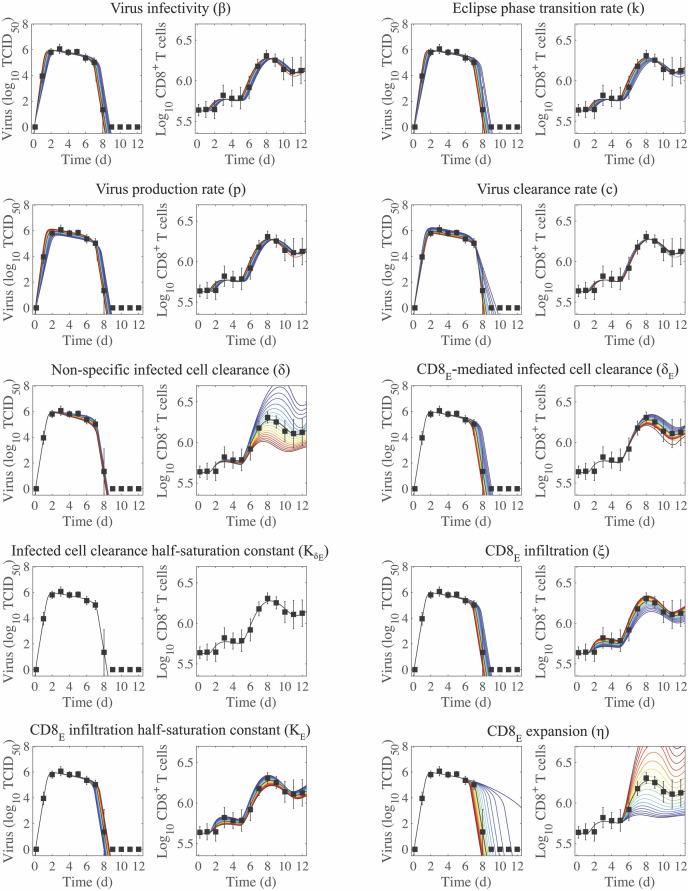
Sensitivity of the CD8^+^ T cell model. Solutions of the CD8^+^ T cell model (Equation (1)-(6)) with the indicated parameter (β, k, p, c, δ, $\delta_{E}$, $K_{\delta_{E}}$, ξ, $K_{E}$, or η) increased (red) or decreased (blue) 50% from the best-fit value (Table 1). CD8_E_ denotes effector CD8^+^ T cells.

**Appendix 3—figure 2.**
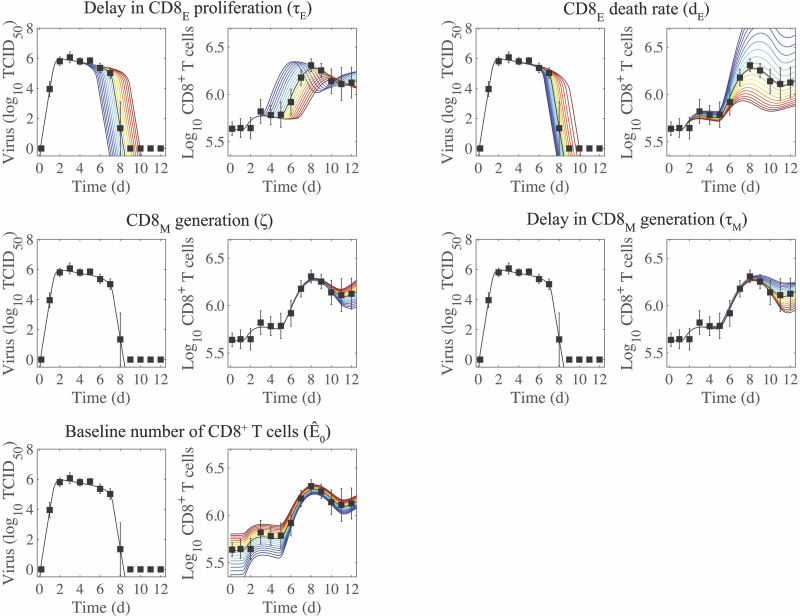
Sensitivity of the CD8^+^ T cell model. Solutions of the CD8^+^ T cell model (Equation (1)-(6)) with the indicated parameter ($\tau_{E}$, $d_{E}$, ζ, $\tau_{M}$, or ${\hat{E}}_{0}$) increased (red) or decreased (blue) 50% from the best-fit value (Table 1). CD8_E_ and CD8_M_ denote effector and memory CD8^+^ T cells, respectively.

## Data Availability

All data generated or analyzed during this study are included in the manuscript and supporting files. Source data files have been provided.

## Additional files

Source data 1. Source data including viral titers, CD8^+^ T cells, inflammation, lesion, and weight loss.

Source code 1. Source code files for reproduction of all figures.

Supplementary file 1. Statistical comparison of alternate models. Comparison of the Akaike Information Criteria (AIC) of the CD8^+^ T cell model in Equation (1)-(6), the alternate model in Equation (A1)-(A6), and the Baral model in Equation (A7)-(A8). The fit of these models is shown in Appendix 1—figure 1.

Supplementary file 2. CD8 T cell depletion model parameters. Parameters, SSR, and AIC_C_ obtained from fitting the CD8^+^ T cell model (Equation (1)-(6)) to viral titers and CD8^+^ T cells from mice infected with 75 TCID_50_ PR8 and with CD8^+^ T cells depleted at −2 d, 0 d, 3 d, and 7 d pi. The total number of CD8^+^ T cells is $\hat{E}=E+E_{M}+\hat{E_{0}}$, where $\hat{E_{0}}=5.6\times 10^{3}\;cells$, and all other parameters are those in Table 1. The best model is bolded.

Transparent reporting form

## Appendix 1

Alternate CD8^+^ T cell Models We examined alternate formulations of the CD8^+^ T cell model to further investigate the density-dependence in the CD8^+^ T cell response. Rather than assuming that CD8_E_-mediated clearance of infected cells is dependent on their density, the model in Equation (A1)-(A6) assumes that the rate of CD8_E_ expansion is dependent on the density of infected cells. Similar models have been used to study CD8^+^ T cell responses during HIV infection (Conway and Perelson, 2015; Bonhoeffer et al., 2000) and other viral infections (Baral et al., 2019). The differences between this alternate model and the CD8^+^ T cell model (Equation (1)-(6)) are in bold.

(A1) $$\frac{d\,T}{d\,t}=-\beta \,T\,V$$

(A2) $$\frac{d\,I_{1}}{d\,t}=\beta \,T\,V-k\,I_{1}$$

(A3) $$\frac{d\,I_{2}}{d\,t}=k\,I_{1}-\delta \,I_{2}-\delta_{\mathrm{𝐄𝐚}}\,{\mathrm{𝐄𝐈}}_{𝟐}$$

(A4) $$\frac{d\,V}{d\,t}=p\,I_{2}-c\,V$$

(A5) $$\frac{d\,E}{d\,t}=\frac{\eta_{\mathrm{𝐄𝐚}}}{{𝐊}_{\mathrm{𝐄𝐚}}+{𝐈}_{𝟐}}\,I_{2}\,\left(t-\tau_{E}\right)-d_{E}\,E$$

(A6) $$\frac{d\,E_{M}}{d\,t}=\zeta \,E\,\left(t-\tau_{M}\right)$$

When a linear CD8_E_-mediated infected cell clearance rate is included, the CD8_E_ dynamics between 3–5 d pi cannot be replicated and returned a statistically worse fit via AIC (Supplementary file 1). However, because these cells may not have effector functions and contribute to infected cell clearance at this time (see Discussion), we excluded these data and the term $\xi \,I_{2}/\left(K_{E}+E\right)$ when fitting Equation (A1)-(A6) to the viral load and total CD8^+^ T cell data (Appendix 1—figure 1). The CD8 dynamics are similar to those generated by CD8^+^ T cell model. However, the alternate model underestimates the data at day seven and the sharp decline between 7–8 d. While it is inappropriate to directly compare these models due to the varying number of data points, we assessed their goodness of fit with and without contributions from the data at 3–5 d pi (Supplementary file 1). Regardless of the data inclusion or exclusion, the alternate model had a higher AIC in all contexts and, thus, is not statistically justifiable. Similarly, we examined the model in Baral et al., 2019 (Equation (A7)-(A8)), which also could not replicate our data (Appendix 1—figure 1).

(A7) $$\frac{d\,I}{d\,t}=\kappa_{b}\,I\,\left(1-\frac{I}{I_{m\,a\,x}}\right)-\delta_{E\,b}\,I\,E$$

(A8) $$\frac{dE}{dt}=\eta_{b}\frac{IE}{K_{Eb}+I}-d_{Eb}\frac{IE}{K_{Db}+I}$$

## Appendix 2

Comparison of the Density-Dependent and CD8^+^ T cell Models We previously developed a density-dependent (DD) viral kinetic model, which describes the biphasic decline of viral loads without inclusion of specific host responses (Smith et al., 2018). This model tracks four populations: susceptible epithelial (‘target’) cells (T), two classes of infected cells ($I_{1}$ and $I_{2}$), and virus (V) (Smith et al., 2018).

(A9) $$\frac{d\,T}{d\,t}=-\beta \,T\,V$$

(A10) $$\frac{d\,I_{1}}{d\,t}=\beta \,T\,V-k\,I_{1}$$

(A11) $$\frac{d\,I_{2}}{d\,t}=k\,I_{1}-\delta_{d}\,\left(I_{2}\right)\,I_{2}$$

(A12) $$\frac{d\,V}{d\,t}=p\,I_{2}-c\,V$$

Briefly, in the DD model, virus-producing infected cells ($I_{2}$) are cleared according to the function $\delta_{d}\,\left(I_{2}\right)=\delta_{d}/\left(K_{\delta }+I_{2}\right)$, where $\delta_{d}/K_{\delta }$ is the maximum per day rate of infected cell clearance and $K_{\delta }$ is the half-saturation constant (Appendix 2—figure 1). All other terms are common to the CD8 T^+^ cell model (Equation (1)-(6)). The DD model provides a close fit to the viral load data in Figure 1B and replicates the biphasic viral load decline while excluding the dynamics of specific immune responses (Smith et al., 2018). Unsurprisingly, the CD8^+^ T cell model is also capable of reproducing the biphasic viral load decay (Figure 1B and Appendix 2—figure 1). In that model, infected cell clearance is split into terms for non-specific clearance (δ) and CD8_E_-mediated clearance ($\delta_{E}\,\left(I_{2},E\right)=\delta_{E}\,E/\left(K_{\delta }+I_{2}\right)$) (Appendix 2—figure 1). Because the CD8^+^ T cell model is more mechanistic than the DD model, most of the correlations between the parameters common to both models (i.e., the rates of virus infectivity (β), virus production (p), and virus clearance (c)) were reduced (Appendix 2—figure 1A). In addition, the correlations between the infected cell clearance parameters ($\delta_{d}$ and $K_{\delta }$ or $\delta_{E}$ and $K_{\delta_{E}}$) and between the rate of virus infectivity (β) and their ratios ($\delta_{d}/K_{\delta }$ or $\delta_{E}/K_{\delta_{E}}$) were abolished (Figure 2—figure supplement 1–2). There was a negative correlation between the infected cell clearance parameters (δ and $\delta_{E}$; Figure 2B), which may reflect the connection between the efficacy of early immune mechanisms and the CD8^+^ T cell response. This result is in line with experimental evidence that the innate immune responses modulate the activation of adaptive immunity (Iwasaki and Medzhitov, 2015; Luster, 2002; Iwasaki and Medzhitov, 2010; Le Bon and Tough, 2002; Jain and Pasare, 2017). The differences in model structure between the two models yielded changes in parameter sensitivity and model behavior during the rapid viral clearance phase (Appendix 2—figure 1). In the DD model, the most sensitive parameter is the infected cell clearance, $\delta_{d}$ (Appendix 2—figure 1). A 50% decrease in this parameter resulted in a ∼7 d delay in viral resolution (Appendix 2—figure 1; Smith et al., 2018). In the CD8^+^ T cell model, however, viral resolution is delayed by <1 d if the CD8_E_-mediated infected cell clearance parameter ($\delta_{E}$) is reduced by 50% (Appendix 2—figure 1, Appendix 3—figure 1). The rates of CD8_E_ expansion (η) and decay ($d_{E}$) are sensitive and, thus, significantly influence the viral resolution kinetics (Figures 1–2). A 50% decrease in η results in a ∼6 d delay in recovery (Appendix 2—figure 1, Appendix 3—figure 1) whereas a 48% decrease in η prolongs the infection by ∼30 d (Figure 3D–E). This bifurcation in recovery time is a unique feature of the CD8^+^ T cell model.

## Appendix 3

Regulation of the CD8^+^ T cell Response To further understand the regulation of the CD8^+^ T cell response, we examined the 2-D parameter ensembles (Figure 2A–C, Figure 2—figure supplements 1–2) and the results from the sensitivity analysis (Appendix 3—figure 1–2). Overall, few parameters were correlated. There was an expected, although small, positive correlation between the rate of CD8_E_ infiltration (ξ) and the associated half-saturation constant ($K_{E}$) (Figure 2—figure supplement 1–2), which represents the coordination between CD8_E_ recruitment and the processes that prevent an overabundance of these cells. Likewise, a negative correlation was detected between the rate of initial CD8_E_ influx (ξ) and the initial number of CD8^+^ T cells (${\hat{E}}_{0}$) (Figure 2—figure supplements 1–2). The influx rate (ξ) was also positively correlated with the delay in CD8_E_ expansion ($\tau_{E}$) (Figure 2—figure supplements 1–2). The rates of CD8_E_ expansion (η) and decay ($d_{E}$) are correlated (Figure 2C), indicating a balance between these two processes. This correlation was expected and reflects the coordination of mechanisms that regulate CD8^+^ T cell numbers, which may be necessary to limit excessive immunopathology while still resolving the infection (Moskophidis and Kioussis, 1998; Duan and Thomas, 2016; La Gruta et al., 2007). Further, because of this correlation and the sensitivity of η (Appendix 3—figure 1), the CD8^+^ T cell kinetics are sensitive to changes in $d_{E}$ (Appendix 3—figure 1). However, increasing the decay rate had less impact on the viral load kinetics, comparatively. Because $d_{E}$ is correlated with both η and the rate of CD8_M_ generation (ζ) (Figure 2C), it naturally follows that η and ζ are correlated (Figure 2—figure supplements 1–2). Changing the rates of virus infectivity (β), production (p), or clearance (c) had little effect on viral load or CD8^+^ T cell kinetics (Appendix 3—figure 1).

## References

[bib1] Ahmed H, Moore J, Manicassamy B, Garcia-Sastre A, Handel A, Antia R (2017). Mathematical analysis of a mouse experiment suggests little role for resource depletion in controlling influenza infection within host. arXiv.

[bib2] Ai T, Yang Z, Hou H, Zhan C, Chen C, Lv W, Tao Q, Sun Z, Xia L (2020). Correlation of chest CT and RT-PCR testing for coronavirus disease 2019 (COVID-19) in China: a report of 1014 cases. Radiology.

[bib3] Ajlan AM, Quiney B, Nicolaou S, Müller NL (2009). Swine-origin influenza A (H1N1) viral infection: radiographic and CT findings. American Journal of Roentgenology.

[bib4] Anderson KG, Sung H, Skon CN, Lefrancois L, Deisinger A, Vezys V, Masopust D (2012). Cutting edge: intravascular staining redefines lung CD8 T cell responses. The Journal of Immunology.

[bib5] Baccam P, Beauchemin C, Macken CA, Hayden FG, Perelson AS (2006). Kinetics of influenza A virus infection in humans. Journal of Virology.

[bib6] Hayden FG, Sugaya N, Hirotsu N, Lee N, de Jong MD, Hurt AC, Ishida T, Sekino H, Yamada K, Portsmouth S, Kawaguchi K, Shishido T, Arai M, Tsuchiya K, Uehara T, Watanabe A, Baloxavir Marboxil Investigators Group (2018). Baloxavir marboxil for uncomplicated influenza in adults and adolescents. New England Journal of Medicine.

[bib7] Baral S, Antia R, Dixit NM (2019). A dynamical motif comprising the interactions between antigens and CD8 T cells may underlie the outcomes of viral infections. PNAS.

[bib8] Bautista E, Chotpitayasunondh T, Gao Z, Harper SA, Shaw M, Uyeki TM, Zaki SR, Hayden FG, Hui DS, Kettner JD, Kumar A, Lim M, Shindo N, Penn C, Nicholson KG, Writing Committee of the WHO Consultation on Clinical Aspects of Pandemic (H1N1) 2009 Influenza (2010). Clinical aspects of pandemic 2009 influenza A (H1N1) virus infection. The New England Journal of Medicine.

[bib9] Beauchemin CA, Handel A (2011). A review of mathematical models of influenza A infections within a host or cell culture: lessons learned and challenges ahead. BMC Public Health.

[bib10] Bedoui S, Gebhardt T (2011). Interaction between dendritic cells and T cells during peripheral virus infections: a role for antigen presentation beyond lymphoid organs?. Current Opinion in Immunology.

[bib11] Bender BS, Croghan T, Zhang L, Small PA (1992). Transgenic mice lacking class I major histocompatibility complex-restricted T cells have delayed viral clearance and increased mortality after influenza virus challenge. Journal of Experimental Medicine.

[bib12] Boianelli A, Nguyen VK, Ebensen T, Schulze K, Wilk E, Sharma N, Stegemann-Koniszewski S, Bruder D, Toapanta FR, Guzmán CA, Meyer-Hermann M, Hernandez-Vargas EA (2015). Modeling influenza virus infection: a roadmap for influenza research. Viruses.

[bib13] Bonhoeffer S, Rembiszewski M, Ortiz GM, Nixon DF (2000). Risks and benefits of structured antiretroviral drug therapy interruptions in HIV-1 infection. Aids.

[bib14] Boon AC, Finkelstein D, Zheng M, Liao G, Allard J, Klumpp K, Webster R, Peltz G, Webby RJ (2011). H5N1 influenza virus pathogenesis in genetically diverse mice is mediated at the level of viral load. mBio.

[bib15] Bouvier NM, Lowen AC (2010). Animal models for influenza virus pathogenesis and transmission. Viruses.

[bib16] Boyd DF, Allen EK, Randolph AG, Guo XJ, Weng Y, Sanders CJ, Bajracharya R, Lee NK, Guy CS, Vogel P, Guan W, Li Y, Liu X, Novak T, Newhams MM, Fabrizio TP, Wohlgemuth N, Mourani PM, Wight TN, Schultz-Cherry S, Cormier SA, Shaw-Saliba K, Pekosz A, Rothman RE, Chen KF, Yang Z, Webby RJ, Zhong N, Crawford JC, Thomas PG, PALISI Pediatric Intensive Care Influenza (PICFLU) Investigators (2020). Exuberant fibroblast activity compromises lung function via ADAMTS4. Nature.

[bib17] Byrd RH, Lu P, Nocedal J, Zhu C (1995). A limited memory algorithm for bound constrained optimization. SIAM Journal on Scientific Computing.

[bib18] Cairns BJ (2008). Pydde.

[bib19] Cao P, Wang Z, Yan AW, McVernon J, Xu J, Heffernan JM, Kedzierska K, McCaw JM (2016). On the role of CD8^+^ T cells in determining recovery time from influenza virus infection. Frontiers in Immunology.

[bib20] Caramalho I, Faroudi M, Padovan E, Müller S, Valitutti S (2009). Visualizing CTL/melanoma cell interactions: multiple hits must be delivered for tumour cell annihilation. Journal of Cellular and Molecular Medicine.

[bib21] Carrat F, Vergu E, Ferguson NM, Lemaitre M, Cauchemez S, Leach S, Valleron AJ (2008). Time lines of infection and disease in human influenza: a review of volunteer challenge studies. American Journal of Epidemiology.

[bib22] Channappanavar R, Fehr AR, Vijay R, Mack M, Zhao J, Meyerholz DK, Perlman S (2016). Dysregulated type I interferon and inflammatory Monocyte-Macrophage responses cause lethal pneumonia in SARS-CoV-Infected mice. Cell Host & Microbe.

[bib23] Chen X, Liu S, Goraya MU, Maarouf M, Huang S, Chen JL (2018). Host immune response to influenza A virus infection. Frontiers in Immunology.

[bib24] Conway JM, Perelson AS (2015). Post-treatment control of HIV infection. PNAS.

[bib25] Cross EW, Blain TJ, Mathew D, Kedl RM (2019). Anti-CD8 monoclonal antibody-mediated depletion alters the phenotype and behavior of surviving CD8+ T cells. PLOS ONE.

[bib26] De Boer RJ, Perelson AS (1995). Towards a general function describing T cell proliferation. Journal of Theoretical Biology.

[bib27] De Boer RJ, Perelson AS (1998). Target cell limited and immune control models of HIV infection: a comparison. Journal of Theoretical Biology.

[bib28] de Jong MD, Simmons CP, Thanh TT, Hien VM, Smith GJ, Chau TN, Hoang DM, Chau NV, Khanh TH, Dong VC, Qui PT, Cam BV, Ha doQ, Guan Y, Peiris JS, Chinh NT, Hien TT, Farrar J (2006). Fatal outcome of human influenza A (H5N1) is associated with high viral load and hypercytokinemia. Nature Medicine.

[bib29] Deguine J, Breart B, Lemaître F, Di Santo JP, Bousso P (2010). Intravital imaging reveals distinct dynamics for natural killer and CD8(+) T cells during tumor regression. Immunity.

[bib30] Dobrovolny HM, Reddy MB, Kamal MA, Rayner CR, Beauchemin CA (2013). Assessing mathematical models of influenza infections using features of the immune response. PLOS ONE.

[bib31] Duan S, Meliopoulos VA, McClaren JL, Guo XZ, Sanders CJ, Smallwood HS, Webby RJ, Schultz-Cherry SL, Doherty PC, Thomas PG (2015). Diverse heterologous primary infections radically alter immunodominance hierarchies and clinical outcomes following H7N9 influenza challenge in mice. PLOS Pathogens.

[bib32] Duan S, Thomas PG (2016). Balancing immune protection and immune pathology by CD8(+) T-Cell responses to influenza infection. Frontiers in Immunology.

[bib33] Dunster JL, Byrne HM, King JR (2014). The resolution of inflammation: a mathematical model of neutrophil and macrophage interactions. Bulletin of Mathematical Biology.

[bib34] Eccles R (2005). Understanding the symptoms of the common cold and influenza. The Lancet Infectious Diseases.

[bib35] Eichelberger M, Allan W, Zijlstra M, Jaenisch R, Doherty PC (1991). Clearance of influenza virus respiratory infection in mice lacking class I major histocompatibility complex-restricted CD8+ T cells. Journal of Experimental Medicine.

[bib36] Fang M, Sigal LJ (2005). Antibodies and CD8+ T cells are complementary and essential for natural resistance to a highly lethal cytopathic virus. The Journal of Immunology.

[bib37] Feikin DR, Fu W, Park DE, Shi Q, Higdon MM, Baggett HC, Brooks WA, Deloria Knoll M, Hammitt LL, Howie SRC, Kotloff KL, Levine OS, Madhi SA, Scott JAG, Thea DM, Adrian PV, Antonio M, Awori JO, Baillie VL, DeLuca AN, Driscoll AJ, Ebruke BE, Goswami D, Karron RA, Li M, Morpeth SC, Mwaba J, Mwansa J, Prosperi C, Sawatwong P, Sow SO, Tapia MD, Whistler T, Zaman K, Zeger SL, O' Brien KL, Murdoch DR, PERCH Study Group (2017). Is higher viral load in the upper respiratory tract associated with severe pneumonia? findings from the PERCH study. Clinical Infectious Diseases.

[bib38] Gadhamsetty S, Marée AF, Beltman JB, de Boer RJ (2014). A general functional response of cytotoxic T lymphocyte-mediated killing of target cells. Biophysical Journal.

[bib39] Gallagher M, Brooke C, Ke R, Koelle K (2018). Causes and consequences of spatial Within-Host viral spread. Viruses.

[bib40] Ganusov VV, Barber DL, De Boer RJ (2011). Killing of targets by CD8 T cells in the mouse spleen follows the law of mass action. PLOS ONE.

[bib41] Gao R, Bhatnagar J, Blau DM, Greer P, Rollin DC, Denison AM, Deleon-Carnes M, Shieh WJ, Sambhara S, Tumpey TM, Patel M, Liu L, Paddock C, Drew C, Shu Y, Katz JM, Zaki SR (2013). Cytokine and chemokine profiles in lung tissues from fatal cases of 2009 pandemic influenza A (H1N1): role of the host immune response in pathogenesis. The American Journal of Pathology.

[bib42] Gill JR, Sheng ZM, Ely SF, Guinee DG, Beasley MB, Suh J, Deshpande C, Mollura DJ, Morens DM, Bray M, Travis WD, Taubenberger JK (2010). Pulmonary pathologic findings of fatal 2009 pandemic influenza A/H1N1 viral infections. Archives of Pathology & Laboratory Medicine.

[bib43] Granados A, Peci A, McGeer A, Gubbay JB (2017). Influenza and rhinovirus viral load and disease severity in upper respiratory tract infections. Journal of Clinical Virology.

[bib44] Graw F, Regoes RR (2009). Investigating CTL mediated killing with a 3D cellular automaton. PLOS Computational Biology.

[bib45] Gubareva LV, Kaiser L, Hayden FG (2000). Influenza virus neuraminidase inhibitors. The Lancet.

[bib46] Halle S, Keyser KA, Stahl FR, Busche A, Marquardt A, Zheng X, Galla M, Heissmeyer V, Heller K, Boelter J, Wagner K, Bischoff Y, Martens R, Braun A, Werth K, Uvarovskii A, Kempf H, Meyer-Hermann M, Arens R, Kremer M, Sutter G, Messerle M, Förster R (2016). In Vivo Killing Capacity of Cytotoxic T Cells Is Limited and Involves Dynamic Interactions and T Cell Cooperativity. Immunity.

[bib47] Han A, Poon JL, Powers JH, Leidy NK, Yu R, Memoli MJ (2018). Using the influenza Patient-reported outcome (FLU-PRO) diary to evaluate symptoms of influenza viral infection in a healthy human challenge model. BMC Infectious Diseases.

[bib48] Handel A, Liao LE, Beauchemin CAA (2018). Progress and trends in mathematical modelling of influenza A virus infections. Current Opinion in Systems Biology.

[bib49] Harrington LE, Galvan M, Baum LG, Altman JD, Ahmed R (2000). Differentiating between memory and effector CD8 T cells by altered expression of cell surface O-glycans. Journal of Experimental Medicine.

[bib50] Holder BP, Beauchemin CA (2011). Exploring the effect of biological delays in kinetic models of influenza within a host or cell culture. BMC Public Health.

[bib51] Hou S, Doherty PC, Zijlstra M, Jaenisch R, Katz JM (1992). Delayed clearance of Sendai virus in mice lacking class I MHC-restricted CD8 T cells. Journal of Immunology.

[bib52] Huang CT, Hung CY, Chen TC, Lin CY, Lin YC, Chang CS, He YC, Huang YL, Dutta A (2017). Rapamycin adjuvant and exacerbation of severe influenza in an experimental mouse model. Scientific Reports.

[bib53] Iwasaki A, Medzhitov R (2010). Regulation of adaptive immunity by the innate immune system. Science.

[bib54] Iwasaki A, Medzhitov R (2015). Control of adaptive immunity by the innate immune system. Nature Immunology.

[bib55] Iwasaki T, Nozima T (1977). Defense mechanisms against primary influenza virus infection in mice: I. the roles of interferon and neutralizing antibodies and Thymus dependence of interferon and antibody production. Journal of Immunology.

[bib56] Jain A, Pasare C (2017). Innate control of adaptive immunity: beyond the Three-Signal paradigm. The Journal of Immunology.

[bib57] Jones AT, Federsppiel B, Ellies LG, Williams MJ, Burgener R, Duronio V, Smith CA, Takei F, Ziltener HJ (1994). Characterization of the activation-associated isoform of CD43 on murine T lymphocytes. Journal of Immunology.

[bib58] Kaech SM, Hemby S, Kersh E, Ahmed R (2002). Molecular and functional profiling of memory CD8 T cell differentiation. Cell.

[bib59] Kang SS, Herz J, Kim JV, Nayak D, Stewart-Hutchinson P, Dustin ML, McGavern DB (2011). Migration of cytotoxic lymphocytes in cell cycle permits local MHC I-dependent control of division at sites of viral infection. Journal of Experimental Medicine.

[bib60] Karlsson EA, Meliopoulos VA, Savage C, Livingston B, Mehle A, Schultz-Cherry S (2015). Visualizing real-time influenza virus infection, transmission and protection in ferrets. Nature Communications.

[bib61] Keating R, Morris MY, Yue W, Reynolds CE, Harris TL, Brown SA, Doherty PC, Thomas PG, McGargill MA (2018). Potential killers exposed: tracking endogenous influenza-specific CD8^+^ T cells. Immunology and Cell Biology.

[bib62] Kohr JR, Bhargava P, Takasugi J, Goodman RB, Medverd JR (2010). Imaging appearance of swine-origin influenza A (novel 2009 H1N1) pneumonia in an immunocompromised patient. Radiology Case Reports.

[bib63] Koshimichi H, Ishibashi T, Kawaguchi N, Sato C, Kawasaki A, Wajima T (2018). Safety, tolerability, and pharmacokinetics of the novel Anti-influenza agent baloxavir marboxil in healthy adults: phase I study findings. Clinical Drug Investigation.

[bib64] Kreijtz JH, Fouchier RA, Rimmelzwaan GF (2011). Immune responses to influenza virus infection. Virus Research.

[bib65] Kugel D, Kochs G, Obojes K, Roth J, Kobinger GP, Kobasa D, Haller O, Staeheli P, von Messling V (2009). Intranasal administration of alpha interferon reduces seasonal influenza A virus morbidity in ferrets. Journal of Virology.

[bib66] La Gruta NL, Kedzierska K, Stambas J, Doherty PC (2007). A question of self-preservation: immunopathology in influenza virus infection. Immunology & Cell Biology.

[bib67] La Gruta NL, Turner SJ (2014). T cell mediated immunity to influenza: mechanisms of viral control. Trends in Immunology.

[bib68] Lambert Emo K, Hyun YM, Reilly E, Barilla C, Gerber S, Fowell D, Kim M, Topham DJ (2016). Live imaging of influenza infection of the Trachea reveals dynamic regulation of CD8+ T cell motility by antigen. PLOS Pathogens.

[bib69] Lauder SN, Taylor PR, Clark SR, Evans RL, Hindley JP, Smart K, Leach H, Kidd EJ, Broadley KJ, Jones SA, Wise MP, Godkin AJ, O'Donnell V, Gallimore AM (2011). Paracetamol reduces influenza-induced immunopathology in a mouse model of infection without compromising virus clearance or the generation of protective immunity. Thorax.

[bib70] Lawrence CW, Ream RM, Braciale TJ (2005). Frequency, specificity, and sites of expansion of CD8+ T cells during primary pulmonary influenza virus infection. The Journal of Immunology.

[bib71] Le D, Miller JD, Ganusov VV (2014). Mathematical modeling provides kinetic details of the human immune response to vaccination. Frontiers in Cellular and Infection Microbiology.

[bib72] Le Bon A, Tough DF (2002). Links between innate and adaptive immunity via type I interferon. Current Opinion in Immunology.

[bib73] Lee HY, Topham DJ, Park SY, Hollenbaugh J, Treanor J, Mosmann TR, Jin X, Ward BM, Miao H, Holden-Wiltse J, Perelson AS, Zand M, Wu H (2009). Simulation and prediction of the adaptive immune response to influenza A virus infection. Journal of Virology.

[bib74] Lee CS, Lee JH (2010). Dynamics of clinical symptoms in patients with pandemic influenza A (H1N1). Clinical Microbiology and Infection.

[bib75] Levin D, Forrest S, Banerjee S, Clay C, Cannon J, Moses M, Koster F (2016). A spatial model of the efficiency of T cell search in the influenza-infected lung. Journal of Theoretical Biology.

[bib76] Li P, Su DJ, Zhang JF, Xia XD, Sui H, Zhao DH (2011). Pneumonia in novel swine-origin influenza A (H1N1) virus infection: high-resolution CT findings. European Journal of Radiology.

[bib77] Li P, Zhang JF, Xia XD, Su DJ, Liu BL, Zhao DL, Liu Y, Zhao DH (2012). Serial evaluation of high-resolution CT findings in patients with pneumonia in novel swine-origin influenza A (H1N1) virus infection. The British Journal of Radiology.

[bib78] Li Y, Handel A (2014). Modeling inoculum dose dependent patterns of acute virus infections. Journal of Theoretical Biology.

[bib79] Lu J, Duan X, Zhao W, Wang J, Wang H, Zhou K, Fang M (2018). Aged mice are more resistant to influenza virus infection due to reduced inflammation and lung pathology. Aging and Disease.

[bib80] Luker KE, Luker GD (2010). Bioluminescence imaging of reporter mice for studies of infection and inflammation. Antiviral Research.

[bib81] Luster AD (2002). The role of chemokines in linking innate and adaptive immunity. Current Opinion in Immunology.

[bib82] Manchanda H, Seidel N, Krumbholz A, Sauerbrei A, Schmidtke M, Guthke R (2014). Within-host influenza dynamics: a small-scale mathematical modeling approach. Biosystems.

[bib83] Manicassamy B, Manicassamy S, Belicha-Villanueva A, Pisanelli G, Pulendran B, García-Sastre A (2010). Analysis of in vivo dynamics of influenza virus infection in mice using a GFP reporter virus. PNAS.

[bib84] Marathe BM, Wong SS, Vogel P, Garcia-Alcalde F, Webster RG, Webby RJ, Najera I, Govorkova EA (2016). Combinations of oseltamivir and T-705 extend the treatment window for highly pathogenic influenza A(H5N1) Virus infection in mice. Scientific Reports.

[bib85] Marathe BM, Mostafa HH, Vogel P, Pascua PNQ, Jones JC, Russell CJ, Webby RJ, Govorkova EA (2017). A pharmacologically immunosuppressed mouse model for assessing influenza B virus pathogenicity and oseltamivir treatment. Antiviral Research.

[bib86] Margine I, Krammer F (2014). Animal models for influenza viruses: implications for universal vaccine development. Pathogens.

[bib87] Matheu MP, Teijaro JR, Walsh KB, Greenberg ML, Marsolais D, Parker I, Rosen H, Oldstone MB, Cahalan MD (2013). Three phases of CD8 T cell response in the lung following H1N1 influenza infection and sphingosine 1 phosphate agonist therapy. PLOS ONE.

[bib88] Mauad T, Hajjar LA, Callegari GD, da Silva LF, Schout D, Galas FR, Alves VA, Malheiros DM, Auler JO, Ferreira AF, Borsato MR, Bezerra SM, Gutierrez PS, Caldini ET, Pasqualucci CA, Dolhnikoff M, Saldiva PH (2010). Lung pathology in fatal novel human influenza A (H1N1) infection. American Journal of Respiratory and Critical Care Medicine.

[bib89] McAuley JL, Hornung F, Boyd KL, Smith AM, McKeon R, Bennink J, Yewdell JW, McCullers JA (2007). Expression of the 1918 influenza A virus PB1-F2 enhances the pathogenesis of viral and secondary bacterial pneumonia. Cell Host & Microbe.

[bib90] McGill J, Legge KL (2009). Cutting edge: contribution of lung-resident T cell proliferation to the overall magnitude of the antigen-specific CD8 T cell response in the lungs following murine influenza virus infection. The Journal of Immunology.

[bib91] McMichael AJ, Gotch FM, Noble GR, Beare PA (1983). Cytotoxic T-cell immunity to influenza. New England Journal of Medicine.

[bib92] Medina RA, García-Sastre A (2011). Influenza A viruses: new research developments. Nature Reviews Microbiology.

[bib93] Memoli MJ, Athota R, Reed S, Czajkowski L, Bristol T, Proudfoot K, Hagey R, Voell J, Fiorentino C, Ademposi A, Shoham S, Taubenberger JK (2014). The natural history of influenza infection in the severely immunocompromised vs nonimmunocompromised hosts. Clinical Infectious Diseases.

[bib94] Mempel TR, Pittet MJ, Khazaie K, Weninger W, Weissleder R, von Boehmer H, von Andrian UH (2006). Regulatory T cells reversibly suppress cytotoxic T cell function independent of effector differentiation. Immunity.

[bib95] Merrill SJ (1982). Foundations of the use of an enzyme-kinetic analogy in cell-mediated cytotoxicity. Mathematical Biosciences.

[bib96] Mestas J, Hughes CC (2004). Of mice and not men: differences between mouse and human immunology. The Journal of Immunology.

[bib97] Miao H, Hollenbaugh JA, Zand MS, Holden-Wiltse J, Mosmann TR, Perelson AS, Wu H, Topham DJ (2010). Quantifying the early immune response and adaptive immune response kinetics in mice infected with influenza A virus. Journal of Virology.

[bib98] Monto AS, Gravenstein S, Elliott M, Colopy M, Schweinle J (2000). Clinical signs and symptoms predicting influenza infection. Archives of Internal Medicine.

[bib99] Moscona A (2005). Neuraminidase inhibitors for influenza. New England Journal of Medicine.

[bib100] Moskophidis D, Kioussis D (1998). Contribution of virus-specific CD8+ cytotoxic T cells to virus clearance or pathologic manifestations of influenza virus infection in a T cell receptor transgenic mouse model. Journal of Experimental Medicine.

[bib101] Müller V, Marée AFM, De Boer RJ (2001). Small variations in multiple parameters account for wide variations in HIV–1 set–points: a novel modelling approach. Proceedings of the Royal Society of London. Series B: Biological Sciences.

[bib102] Music N, Reber AJ, Lipatov AS, Kamal RP, Blanchfield K, Wilson JR, Donis RO, Katz JM, York IA (2014). Influenza vaccination accelerates recovery of ferrets from lymphopenia. PLOS ONE.

[bib103] Onami TM, Harrington LE, Williams MA, Galvan M, Larsen CP, Pearson TC, Manjunath N, Baum LG, Pearce BD, Ahmed R (2002). Dynamic regulation of T cell immunity by CD43. Journal of Immunology.

[bib104] Ostler T, Davidson W, Ehl S (2002). Virus clearance and immunopathology by CD8(+) T cells during infection with respiratory syncytial virus are mediated by IFN-gamma. European Journal of Immunology.

[bib105] Parzych EM, DiMenna LJ, Latimer BP, Small JC, Kannan S, Manson B, Lasaro MO, Wherry EJ, Ertl HC (2013). Influenza virus specific CD8⁺ T cells exacerbate infection following high dose influenza challenge of aged mice. BioMed Research International.

[bib106] Perelson AS, Bell GI (1982). Delivery of lethal hits by cytotoxic T lymphocytes in multicellular conjugates occurs sequentially but at random times. Journal of Immunology.

[bib107] Perry M (2018). Simanneal.

[bib108] Pilyugin SS, Antia R (2000). Modeling immune responses with handling time. Bulletin of Mathematical Biology.

[bib109] Price I, Mochan-Keef ED, Swigon D, Ermentrout GB, Lukens S, Toapanta FR, Ross TM, Clermont G (2015). The inflammatory response to influenza A virus (H1N1): An experimental and mathematical study. Journal of Theoretical Biology.

[bib110] Ramirez-Zuniga I, Rubin JE, Swigon D, Clermont G (2019). Mathematical modeling of energy consumption in the acute inflammatory response. Journal of Theoretical Biology.

[bib111] Reed LJ, Muench H (1938). A simple method of estimating fifty per cent endpoints12. American Journal of Epidemiology.

[bib112] Reynolds A, Rubin J, Clermont G, Day J, Vodovotz Y, Bard Ermentrout G (2006). A reduced mathematical model of the acute inflammatory response: I. Derivation of model and analysis of anti-inflammation. Journal of Theoretical Biology.

[bib113] Russell DG, Cardona PJ, Kim MJ, Allain S, Altare F (2009). Foamy macrophages and the progression of the human tuberculosis granuloma. Nature Immunology.

[bib114] Ruuskanen O, Lahti E, Jennings LC, Murdoch DR (2011). Viral pneumonia. Lancet.

[bib115] Rygiel TP, Rijkers ES, de Ruiter T, Stolte EH, van der Valk M, Rimmelzwaan GF, Boon L, van Loon AM, Coenjaerts FE, Hoek RM, Tesselaar K, Meyaard L (2009). Lack of CD200 enhances pathological T cell responses during influenza infection. The Journal of Immunology.

[bib116] Sartorius A, Lu Q, Vieira S, Tonnellier M, Lenaour G, Goldstein I, Rouby JJ (2007). Mechanical ventilation and lung infection in the genesis of air-space enlargement. Critical Care.

[bib117] Shieh WJ, Blau DM, Denison AM, Deleon-Carnes M, Adem P, Bhatnagar J, Sumner J, Liu L, Patel M, Batten B, Greer P, Jones T, Smith C, Bartlett J, Montague J, White E, Rollin D, Gao R, Seales C, Jost H, Metcalfe M, Goldsmith CS, Humphrey C, Schmitz A, Drew C, Paddock C, Uyeki TM, Zaki SR (2010). 2009 pandemic influenza A (H1N1): pathology and pathogenesis of 100 fatal cases in the United States. The American Journal of Pathology.

[bib118] Simonsen L, Fukuda K, Schonberger LB, Cox NJ (2000). The impact of influenza epidemics on hospitalizations. The Journal of Infectious Diseases.

[bib119] Slütter B, Van Braeckel-Budimir N, Abboud G, Varga SM, Salek-Ardakani S, Harty JT (2017). Dynamics of influenza-induced lung-resident memory T cells underlie waning heterosubtypic immunity. Science Immunology.

[bib120] Smith AM, Adler FR, Perelson AS (2010). An accurate two-phase approximate solution to an acute viral infection model. Journal of Mathematical Biology.

[bib121] Smith AM, Adler FR, McAuley JL, Gutenkunst RN, Ribeiro RM, McCullers JA, Perelson AS (2011a). Effect of 1918 PB1-F2 expression on influenza A virus infection kinetics. PLOS Computational Biology.

[bib122] Smith AM, McCullers JA, Adler FR (2011b). Mathematical model of a three-stage innate immune response to a pneumococcal lung infection. Journal of Theoretical Biology.

[bib123] Smith AM, Adler FR, Ribeiro RM, Gutenkunst RN, McAuley JL, McCullers JA, Perelson AS (2013). Kinetics of coinfection with influenza A virus and *Streptococcus pneumoniae*. PLOS Pathogens.

[bib124] Smith AP, Moquin DJ, Bernhauerova V, Smith AM (2018). Influenza Virus Infection Model With Density Dependence Supports Biphasic Viral Decay. Frontiers in Microbiology.

[bib125] Smith AM (2018a). Validated models of immune response to virus infection. Current Opinion in Systems Biology.

[bib126] Smith AM (2018b). Host-pathogen kinetics during influenza infection and coinfection: insights from predictive modeling. Immunological Reviews.

[bib127] Smith CA, Kulkarni U, Chen J, Goldstein DR (2019). Influenza virus inoculum volume is critical to elucidate age-dependent mortality in mice. Aging Cell.

[bib128] Smith AM, McCullers JA (2014). Secondary bacterial infections in influenza virus infection pathogenesis. Current Topics in Microbiology and Immunology.

[bib129] Smith AM, Perelson AS (2011). Influenza A virus infection kinetics: quantitative data and models. Wiley Interdisciplinary Reviews: Systems Biology and Medicine.

[bib130] Soto-Abraham MV, Soriano-Rosas J, Díaz-Quiñónez A, Silva-Pereyra J, Vazquez-Hernandez P, Torres-López O, Roldán A, Cruz-Gordillo A, Alonso-Viveros P, Navarro-Reynoso F (2009). Pathological changes associated with the 2009 H1N1 virus. New England Journal of Medicine.

[bib131] Srivastava B, Błazejewska P, Hessmann M, Bruder D, Geffers R, Mauel S, Gruber AD, Schughart K (2009). Host genetic background strongly influences the response to influenza a virus infections. PLOS ONE.

[bib132] Sun J, Madan R, Karp CL, Braciale TJ (2009). Effector T cells control lung inflammation during acute influenza virus infection by producing IL-10. Nature Medicine.

[bib133] Szretter KJ, Gangappa S, Belser JA, Zeng H, Chen H, Matsuoka Y, Sambhara S, Swayne DE, Tumpey TM, Katz JM (2009). Early control of H5N1 influenza virus replication by the type I interferon response in mice. Journal of Virology.

[bib134] Tang BM, Shojaei M, Teoh S, Meyers A, Ho J, Ball TB, Keynan Y, Pisipati A, Kumar A, Eisen DP, Lai K, Gillett M, Santram R, Geffers R, Schreiber J, Mozhui K, Huang S, Parnell GP, Nalos M, Holubova M, Chew T, Booth D, Kumar A, McLean A, Schughart K (2019). Neutrophils-related host factors associated with severe disease and fatality in patients with influenza infection. Nature Communications.

[bib135] Taubenberger JK, Morens DM (2008). The pathology of influenza virus infections. Annual Review of Pathology: Mechanisms of Disease.

[bib136] Thangavel RR, Bouvier NM (2014). Animal models for influenza virus pathogenesis, transmission, and immunology. Journal of Immunological Methods.

[bib137] Thompson WW, Shay DK, Weintraub E, Brammer L, Bridges CB, Cox NJ, Fukuda K (2004). Influenza-associated hospitalizations in the united states. Jama.

[bib138] Toapanta FR, Ross TM (2009). Impaired immune responses in the lungs of aged mice following influenza infection. Respiratory Research.

[bib139] Toniolo Neto J (2014). Neuraminidase inhibitors for preventing and treating influenza in healthy adults and children. Sao Paulo Medical Journal.

[bib140] Trammell RA, Toth LA (2011). Markers for predicting death as an outcome for mice used in infectious disease research. Comparative Medicine.

[bib141] Tran V, Moser LA, Poole DS, Mehle A (2013). Highly sensitive real-time in vivo imaging of an influenza reporter virus reveals dynamics of replication and spread. Journal of Virology.

[bib142] Treanor JJ, Hayden FG, Vrooman PS, Barbarash R, Bettis R, Riff D, Singh S, Kinnersley N, Ward P, Mills RG (2000). Efficacy and safety of the oral neuraminidase inhibitor oseltamivir in treating acute influenza: a randomized controlled trial. US Oral Neuraminidase Study Group. JAMA.

[bib143] Uddbäck I, Cartwright EK, Schøller AS, Wein AN, Hayward SL, Lobby J, Takamura S, Thomsen AR, Kohlmeier JE, Christensen JP (2021). Long-term maintenance of lung resident memory T cells is mediated by persistent antigen. Mucosal Immunology.

[bib144] Ulrichs T, Kosmiadi GA, Trusov V, Jörg S, Pradl L, Titukhina M, Mishenko V, Gushina N, Kaufmann SH (2004). Human tuberculous granulomas induce peripheral lymphoid follicle-like structures to orchestrate local host defence in the lung. The Journal of Pathology.

[bib145] Van Braeckel-Budimir N, Varga SM, Badovinac VP, Harty JT (2018). Repeated Antigen Exposure Extends the Durability of Influenza-Specific Lung-Resident Memory CD8^+^ T Cells and Heterosubtypic Immunity. Cell Reports.

[bib146] van de Sandt CE, Bárcena M, Koster AJ, Kasper J, Kirkpatrick CJ, Scott DP, de Vries RD, Herold S, Rimmelzwaan GF, Kuiken T, Short KR (2017). Human CD8^+^ T Cells Damage Noninfected Epithelial Cells during Influenza Virus Infection In Vitro. American Journal of Respiratory Cell and Molecular Biology.

[bib147] Wakim LM, Smith J, Caminschi I, Lahoud MH, Villadangos JA (2015). Antibody-targeted vaccination to lung dendritic cells generates tissue-resident memory CD8 T cells that are highly protective against influenza virus infection. Mucosal Immunology.

[bib148] Wang Z, Wan Y, Qiu C, Quiñones-Parra S, Zhu Z, Loh L, Tian D, Ren Y, Hu Y, Zhang X, Thomas PG, Inouye M, Doherty PC, Kedzierska K, Xu J (2015). Recovery from severe H7N9 disease is associated with diverse response mechanisms dominated by CD8⁺ T cells. Nature Communications.

[bib149] Watanabe S, Alexander M, Misharin AV, Budinger GRS (2019). The role of macrophages in the resolution of inflammation. The Journal of Clinical Investigation.

[bib150] Weinfurter JT, Brunner K, Capuano SV, Li C, Broman KW, Kawaoka Y, Friedrich TC (2011). Cross-reactive T cells are involved in rapid clearance of 2009 pandemic H1N1 influenza virus in nonhuman primates. PLOS Pathogens.

[bib151] Wells MA, Albrecht P, Ennis FA (1981). Recovery from a viral respiratory infection. I. Influenza pneumonia in normal and T-deficient mice. Journal of Immunology.

[bib152] Wiedemann A, Depoil D, Faroudi M, Valitutti S (2006). Cytotoxic T lymphocytes kill multiple targets simultaneously via spatiotemporal uncoupling of lytic and stimulatory synapses. PNAS.

[bib153] Wilkinson TM, Li CK, Chui CS, Huang AK, Perkins M, Liebner JC, Lambkin-Williams R, Gilbert A, Oxford J, Nicholas B, Staples KJ, Dong T, Douek DC, McMichael AJ, Xu XN (2012). Preexisting influenza-specific CD4+ T cells correlate with disease protection against influenza challenge in humans. Nature Medicine.

[bib154] Wong HYF, Lam HYS, Fong AH, Leung ST, Chin TW, Lo CSY, Lui MM, Lee JCY, Chiu KW, Chung TW, Lee EYP, Wan EYF, Hung IFN, Lam TPW, Kuo MD, Ng MY (2020). Frequency and Distribution of Chest Radiographic Findings in Patients Positive for COVID-19. Radiology.

[bib155] Wu H, Kumar A, Miao H, Holden-Wiltse J, Mosmann TR, Livingstone AM, Belz GT, Perelson AS, Zand MS, Topham DJ (2011). Modeling of influenza-specific CD8+ T cells during the primary response indicates that the spleen is a major source of effectors. Journal of Immunology.

[bib156] Xu L, Yoon H, Zhao MQ, Liu J, Ramana CV, Enelow RI (2004). Cutting edge: pulmonary immunopathology mediated by antigen-specific expression of TNF-alpha by antiviral CD8+ T cells. Journal of Immunology.

[bib157] Xue KS, Stevens-Ayers T, Campbell AP, Englund JA, Pergam SA, Boeckh M, Bloom JD (2017). Parallel evolution of influenza across multiple spatiotemporal scales. eLife.

[bib158] Yannelli JR, Sullivan JA, Mandell GL, Engelhard VH (1986). Reorientation and fusion of cytotoxic T lymphocyte granules after interaction with target cells as determined by high resolution cinemicrography. Journal of Immunology.

[bib159] Yap KL, Ada GL (1978). Cytotoxic T cells in the lungs of mice infected with an influenza A virus. Scandinavian Journal of Immunology.

[bib160] Yates AJ, Van Baalen M, Antia R (2011). Virus replication strategies and the critical CTL numbers required for the control of infection. PLOS Computational Biology.

[bib161] Yoon H, Legge KL, Sung SS, Braciale TJ (2007). Sequential activation of CD8+ T cells in the draining lymph nodes in response to pulmonary virus infection. Journal of Immunology.

[bib162] Zachariadis O, Cassidy JP, Brady J, Mahon BP (2006). gammadelta T cells regulate the early inflammatory response to bordetella pertussis infection in the murine respiratory tract. Infection and Immunity.

[bib163] Zagury D, Bernard J, Thierness N, Feldman M, Berke G (1975). Isolation and characterization of individual functionally reactive cytotoxic T lymphocytes: conjugation, killing and recycling at the single cell level. European Journal of Immunology.

[bib164] Zens KD, Chen JK, Farber DL (2016). Vaccine-generated lung tissue–resident memory T cells provide heterosubtypic protection to influenza infection. JCI Insight.

[bib165] Zhang N, Bevan MJ (2011). CD8(+) T cells: foot soldiers of the immune system. Immunity.

[bib166] Zhu C, Byrd RH, Lu P, Nocedal J (1997). Algorithm 778: L-BFGS-B: Fortran subroutines for large-scale bound-constrained optimization. ACM T Math Software.

