# Supplementary material for: Dynamically linking influenza virus infection kinetics, lung injury, inflammation, and disease severity
Source: eLife. 2021 Jul 20;10:e68864. doi: 10.7554/eLife.68864 (PMC8370774; doi:10.7554/eLife.68864)
Supplement: Supplementary file 1. — Comparison of the Akaike Information Criteria (AIC) of the CD8+ T cell model in Equation (1)-(6), the alternate model in Equation (A1)-(A6), and the Baral model in Equation (A7)-(A8). The fit of these models is shown in Appendix 1—figure 1. [file elife-68864-supp1.pdf]

Supplementary file 1

Statistical comparison of alternate models. Comparison of the Akaike Information Criteria (AIC) of the $CD8^+$ T cell model in Equations (1)–(6), the alternate model in Appendix 1-Equations (1)–(6), and the Baral model in Appendix 1-Equations (7)–(8). The fit of these models is shown in Appendix 1 Figure 1.

	**Total CD8**		**Lung CD8**	
	**All data**	**Excl. d3-5**	**All data**	**Excl. d3-5**
$CD8^+$ T cell Model (Eqns (1)–(6))	32.1	26.0	77.9	63.2
Alternate Model (Appendix 1-Eqns (1)–(6))	69.5	64.9	84.7	72.5
Baral Model (Appendix 1-Eqns (7)–(8))	151.9	135.2	118.2	103.2
