# Supplementary material for: Dynamically linking influenza virus infection kinetics, lung injury, inflammation, and disease severity
Source: eLife. 2021 Jul 20;10:e68864. doi: 10.7554/eLife.68864 (PMC8370774; doi:10.7554/eLife.68864)
Supplement: Supplementary file 2. — Parameters, SSR, and AICC obtained from fitting the CD8+ T cell model (Equation (1)-(6)) to viral titers and CD8+ T cells from mice infected with 75 TCID50 PR8 and with CD8+ T cells depleted at −2 d, 0 d, 3 d, and 7 d pi. The total number of CD8+ T cells is E^=E+EM+E0^, where E0^=5.6×10^3^ cells, and all other parameters are those in Table 1. The best model is bolded. [file elife-68864-supp2.pdf]

Supplementary file 2

$CD8^+$ T cell depletion model parameters. Parameters, SSR, and AIC_c obtained from fitting the $CD8^+$ T cell model (Equations (1)–(6)) to viral titers and $CD8^+$ T cells from mice infected with 75 $TCID_{50}$ PR8 and with $CD8^+$ T cells depleted at -2, 0, 3, and 7 d pi. The total number of $CD8^+$ T cells is $\hat{E} = E + E_M + \hat{E}_0$, where $\hat{E}_0 = 5.6e3$ cells, and all other parameters are those in Table 1. The best model is bolded.

T_0	p	δ	c	β	δ_E	ξ	η	d_E	K_δ	τ_E	**SSR**	**AIC_c**
3.17e6											342.34	60.86
	0.39										320.36	60.19
		1.09									177.95	54.31
			34.95								295.88	59.40
				1.29e-4							62.93	43.92
					2.63						52.72	42.15
						3.81e4					63.13	43.95
							2.81e-7				52.24	42.06
								0.84			53.51	42.30
									3.26e-8		346.68	60.98
										3.20	60.74	43.57
3.57e6							9.91e-7				5.39	22.57
				2.24e-5	6.04						18.40	34.84
		0.45					3.58e-7				20.11	35.72
		0.42			5.19						20.53	35.93
						4.29e3	4.14e-7				25.72	38.19
7.97e6					4.48						48.50	44.53
		0.22			2.63						52.54	45.33
1.00e7						3.28e4					61.30	46.87
**3.98e6**						**1.33e4**	**1.01e-6**				**2.44**	**18.92**
			27.88			6.20e3	4.21e-7				7.33	29.92
	0.51					1.04e4	4.15e-7				15.22	37.23
	0.40	0.14			5.55						16.46	38.01
		0.34				1.85e3	5.00e-7				17.39	38.56
				2.21e-05	7.98	1.95e4					17.45	38.59
	0.47	0.28					3.55e-7				18.20	39.02
		0.23		2.24e-5	6.08						18.38	39.11
	0.44	0.26				8.10e4					44.31	47.91
	0.48	0.37				4.57e3	5.18e-7				2.40	24.75
		0.44		2.54e-5		5.92e3	5.44e-7				4.35	30.70
3.62e6		0.24			1.63		1.01e-6				4.80	31.69
